# Healthy lifestyles and better periodontal health: Results from two large population‐based surveys

**DOI:** 10.1111/jre.13320

**Published:** 2024-07-02

**Authors:** Crystal Marruganti, Shailly Luthra, Syed Basit Hussain, Jeanie Suvan, Francesco D'Aiuto

**Affiliations:** ^1^ Periodontology Unit, Eastman Dental Institute University College London London UK; ^2^ Department of Medical Biotechnologies University of Siena Siena Italy; ^3^ University of Glasgow Dental School Glasgow UK

**Keywords:** activity, physical, epidemiology, lifestyles, periodontal diseases, systemic inflammation

## Abstract

**Aim:**

To ascertain whether healthy lifestyles are associated with periodontal diseases in two large‐scale surveys in the US (National Health and Nutrition Examination Survey – NHANES) and the UK Biobank.

**Methods:**

9854 US adults and 111 679 UK adults were included in the analyses. A healthy lifestyle score (HLS), ranging between 0 and 5, was calculated based on the reported number of healthy behaviours, including never smoking, no heavy alcohol consumption, top third of leisure‐time physical activity, higher dietary quality, and ideal sleep duration. The prevalence of periodontal diseases was the primary outcome in both surveys. In the NHANES, periodontal status was assessed through a full‐mouth periodontal examination, while in the UKB, only self‐reported periodontal status was available.

**Results:**

Multiple regression analyses confirmed that the presence of at least 2–3 healthy behaviours (vs. 0–1) was associated with lower odds of overall and severe periodontitis (ORs 0.5, 0.4–0.6; *p* < .001 and 0.5, 0.3–0.8; *p* = .003, respectively) in the NHANES, and of bleeding gums (OR = 0.9, 0.8–1.0; *p* = .092) and loose teeth (OR = 0.6, 0.5–0.7; *p* < .001) in UKB. This association increased when considering prevalence of 4–5 healthy behaviours (vs. 0–1) in both the NHANES (periodontitis: OR = 0.3, 0.2–0.4; *p* < .001; severe periodontitis: OR = 0.1, 0.01–0.2; *p* < .001) and the UKB (bleeding gums: OR = 0.8, 0.7–0.9; *p* < .001; loose teeth: OR = 0.5, 0.4–0.6; *p* < .001). Mediation analyses revealed how these protective associations could be partially mediated (1–14%) by differences in biomarkers of systemic inflammation (white blood cells and neutrophils count as well as C‐reactive protein).

**Conclusions:**

Adoption of healthy lifestyle behaviours is associated with a lower prevalence of periodontal diseases within two large population‐based samples. This relationship exhibits a dose–response pattern, implying that greater adherence to healthy habits leads to a more significant protective effect against the odds of periodontal diseases. Additionally, our findings suggest that this protective effect is, in part, mediated by reductions in systemic inflammation.

## Graphical abstract

Healthy lifestyle behaviours are associated with a lower prevalence of periodontal diseases in two large scale US and UK populations in a dose‐response manner.

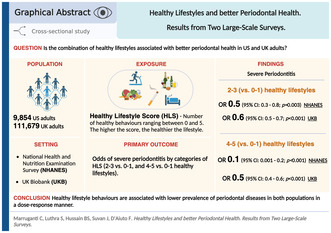

## 1 INTRODUCTION

Periodontal diseases are a group of chronic inflammatory, non‐communicable diseases (NCD) affecting the tooth‐supporting tissues; they comprise both gingivitis, the reversible state of gingival inflammation, and periodontitis, which is characterized by the irreversible loss of the bone supporting the teeth.^1^ According to the Global Burden of Disease Study (GBD; 1990–2010), severe periodontitis is the 7th most prevalent condition worldwide, with around 1.1 billion people being affected by the severe form of the disease, and a global burden increasing by 67.9% between 1990 and 2019.^2^ Individuals affected by severe periodontitis eventually experience tooth loss, hence leading to severe masticatory dysfunction, poor nutrition, edentulism, low quality of life, and self‐esteem.^3^, ^4^ The staggeringly high prevalence of periodontitis and its complications end up imposing a massive socio‐economic impact, as well as hefty healthcare costs. In 2010, it was estimated that the global cost of lost productivity from severe periodontitis was around 54 billion USD/year and that the majority of the 442 billion USD of direct and indirect costs of oral diseases was due to periodontitis.^1^, ^4^ Given that the prevalence of periodontitis increases with age, the overall burden of periodontal diseases is expected to rise dramatically.^1^, ^5^

Periodontal diseases share multiple risk factors with other NCDs, including heart disease, diabetes, and cancer, among others.^6^, ^7^, ^8^ In particular, unhealthy lifestyle behaviours (e.g., smoking, poor nutrition, physical inactivity) are at the root of the global burden of many NCDs and multimorbidities.^9^ Over the past two decades, several studies have highlighted the detrimental impact of some of these behaviours on the onset and progression of periodontal diseases.^10^, ^11^, ^12^, ^13^ For example, a high diet quality, identified as the pattern of consumption of a set of foods in line with key international nutritional guidelines, and high leisure‐time physical exercise were associated with significantly lower odds of periodontal diseases.^14^, ^15^

Recent evidence has however focused on the combination of healthy/unhealthy lifestyles as key determinant in the development of future morbidity and mortality.^16^ The importance of investigating the whole lifestyle (instead of considering each factor separately) relies on the close interconnection among behaviours and on the ability of one behaviour to trigger other adaptive changes,^17^ hence hampering an accurate evaluation of the impact of each separate component on overall health. Evidence on the association between combinations of lifestyles and periodontal diseases is still limited. The aim of the present study was therefore to ascertain whether healthy lifestyles are associated with periodontal diseases in two large‐scale surveys, the National Health and Nutrition Examination Survey – NHANES in the US and the UK Biobank – UKB.

## 2 MATERIALS AND METHODS

This cross‐sectional study was conducted on two populations and was reported according to the STROBE guidelines.^18^

### 2.1 Study samples

Data for the present study were drawn from two large‐scale epidemiological surveys:

1. NHANES (2009–2014 cycles): a nationwide, stratified, multistage probability survey representative of the civilian non‐institutionalized US population. Detailed information about the survey design is provided elsewhere^19^;
2. UKB (2007–2010): a non‐representative sample of the UK population recruited as part of a nationwide epidemiological study carried out between 2006 and 2010 in 22 assessment centres throughout the UK. Detailed information about the study design is provided elsewhere.^20^

### 2.2 Lifestyles assessment

In both surveys, participants were included in the analyses if a complete assessment for all the following healthy lifestyle behaviours was available (Appendix S1):

- Non‐smoking (vs. smoking) (NHANES/UKB);
- Alcohol below the recommended limitations (vs. above), corresponding to 2 alcoholic drinks/day for men and 1 alcoholic drink/day for women (NHANES/UKB)^21^;
- High leisure‐time physical activity (LTPA; vs. low), if the participant was in the top third of the total leisure‐time physical activity level^22^ (NHANES/UKB);
- High diet quality (vs. low), if the participant was in the top two quintiles of the Healthy Eating Index (HEI) for NHANES,^23^ or if the participant had an ideal intake of at least 5 out of the 10 dietary components considered in UKB (i.e., increased consumption of fruits, vegetables, whole grains, (shell)fish, dairy products, and vegetable oils; and reduced or no consumption of refined grains, (un)processed meats and sugar‐sweetened beverages^22^);
- Ideal sleep duration (vs. insufficient/excessive) was defined if the participant reported sleeping 7–9 h and was <65 years, or 7–8 h and was ≥65 years^24^ (NHANES/UKB).

In both databases, all lifestyle behaviours were self‐assessed and self‐reported. Afterward, a healthy lifestyle score (HLS) was then calculated considering the reported number of healthy lifestyle behaviours. The score ranged between 0 and 5, with the highest value indicating the healthiest overall lifestyle. In the analyses, the HLS was categorized as 0–1 vs. 2–3 vs. 4–5 healthy lifestyles, considering 0–1 as the reference category as previously reported.^22^

### 2.3 Periodontal diseases assessment

In the NHANES, all survey participants aged at least 30 years, presenting at least one tooth (excluding third molars) were eligible for a full‐mouth periodontal examination performed by trained and calibrated examiners.^19^, ^25^ Periodontitis case definition was based on those previously reported by the AAP/CDC criteria.^26^

In the UKB, participants reported the presence of any periodontal diseases through a questionnaire. Bleeding gums and painful gums were used as surrogates for mild to moderate periodontal diseases, while self‐reported loose teeth were indicative of severe periodontal diseases.^27^ In cases of multiple responses, the most severe indicator was used as the primary surrogate for periodontal diseases.

### 2.4 Confounders

A number of variables including age, gender, ethnicity, income, acculturation score (a three‐point score based on the country of birth and length of time in the US/UK),^22^ education, body mass index, frequency of self‐performed oral hygiene (NHANES), last dental visit (NHANES), and the number of comorbidities (comorbidity score) were tested as putative confounders using univariate analysis. In both datasets, the comorbidity score ranged between 0 and 4, based on the number of diagnoses among diabetes, hypertension, cardiovascular/cerebrovascular diseases, and depressive symptoms (Appendix S2). Each confounder was tested for its association with the outcome using simple regression analysis. Whenever a confounder was associated both with the exposure and the outcome, it was selected to be included in the multiple models. A detailed description of confounders’ assessment methods was reported in Appendix S2.

### 2.5 Mediators

Potential mediators comprised biomarkers of systemic inflammation, including white blood cells (WBC) and neutrophils counts (1000 cells/μL), and C‐reactive protein (mg/dL) (Appendix S3).

### 2.6 Statistical analyses

All analyses were performed setting the level of significance at 5% (STATA BE, version 17.1, StataCorp LP). For NHANES data, analyses for complex samples were used. Continuous variables were expressed as mean (linearized standard error, SE, for NHANES, and standard deviation, SD, for UKB), while categorical variables were presented as proportions (SE) for NHANES, and as number of observations (percentage, %) for UKB. Participants' characteristics were compared across subgroups of HLS using parametric or non‐parametric tests according to their distribution. The prevalence of periodontal diseases with 95% Confidence Intervals (CI) according to categories of HLS was then plotted for both surveys. Firstly, simple and multiple linear/logistic regression models were built to evaluate the crude and adjusted estimates of the association between each single lifestyle (smoking, alcohol, physical activity, diet quality, sleep duration), the HLS (0–1 vs. 2–3; 0–1 vs. 4–5 healthy lifestyles), and the different measures of periodontal status. All analyses were stratified by age group (<60 vs. ≥60 years)^28^ and sex (males vs. females). Sensitivity analyses were performed in systemically healthy individuals, across the different BMI categories (underweight, normal weight, overweight, and obese) and according to last dental visit (6 months or less, more than 6 months), as well as the frequency of interproximal brushing (0 days/week, 1–6 days/week, 7 days/week). Mediation analysis was performed to ascertain the effect of systemic inflammation on the link between lifestyles and periodontal diseases. Two different and prespecified routes were used; direct (route 1) and indirect (route 2) mediation effects with 95% CI were estimated: route 1: HLS (exposure) ➔ periodontitis (outcome); route 2: HLS (exposure) ➔ systemic inflammatory markers (mediators) ➔ periodontitis (outcome). A propensity score‐matched (PSM) sample was then generated using a 1:1 ratio and the coefficient of mediation analysis for each factor was also calculated using the PSM. Correlation tests were also run to study the association between markers of systemic inflammation and periodontal diseases in both cohorts.

## 3 RESULTS

**FIGURE 1:**
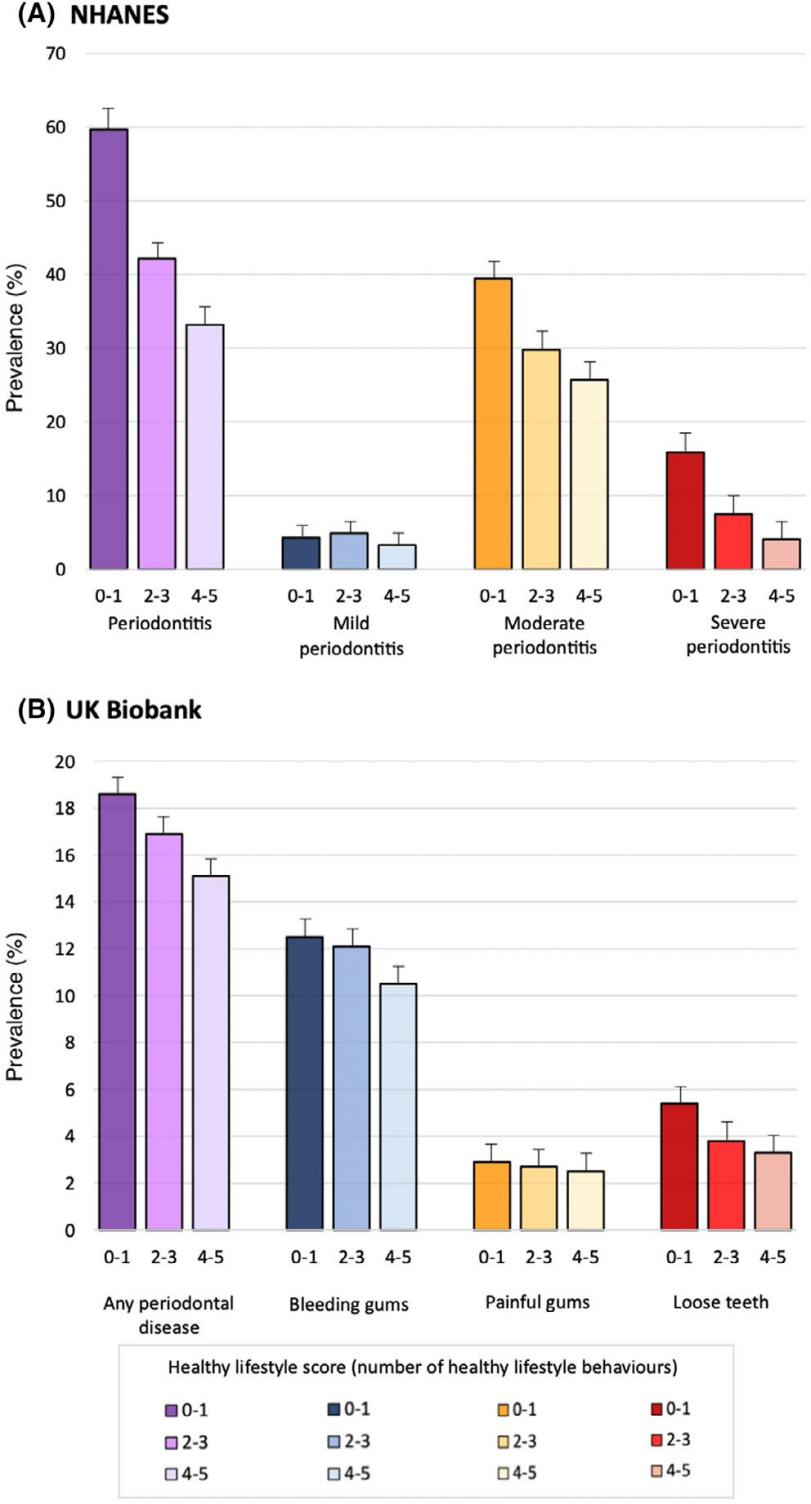
Prevalence of periodontitis according to categories of healthy lifestyle score (error bars indicate 95% Confidence Interval) in the NHANES (A) and UKB (B) databases.

The final datasets included 9854 participants for the NHANES and 111 679 participants for the UKB with complete lifestyles and periodontal assessments (Tables 1 and 2) (missing data, Table S1). The average age of participants was slightly higher in the UKB compared to the NHANES database (55.7 vs. 50.9 years), while the distribution of genders and smoking habits was comparable (Tables 1 and 2). Participants’ characteristics were comparable across subgroups of HLS, except for age in the NHANES dataset (Table 1). The prevalence of periodontal diseases was evaluated according to categories of HLS (Figure 1).

**TABLE 1:** Characteristics of the study population in the NHANES cohort.

Variables	Overall (*N* = 9954)	Healthy lifestyle score			
		0–1 (1662)	2–3 (5535)	4–5 (2657)	*p*‐Value
Age (years), mean (SE)	50.9 (0.3)	47.2 (0.4)	51.1 (0.3)	53.2 (0.5)	.*037* *
*Gender*, *%* (*SE*)					
Male	48.9 (0.005)	55.4 (0.01)	49.5 (0.008)	47.3 (0.01)	.412
Female	51.2 (0.005)	44.7 (0.01)	50.5 (0.008)	52.7 (0.01)	
*Ethnicity*, *%* (*SE*)					
Non‐hispanic Black	10.7 (0.009)	14.7 (0.02)	11.4 (0.01)	6.2 (0.006)	.213
Non‐hispanic White	68.4 (0.02)	66.4 (0.02)	67.1 (0.02)	75.7 (0.01)	
Mexican	8.1 (0.01)	8.5 (0.01)	9.2 (0.01)	5.3 (0.008)	
Other	12.8 (0.009)	10.3 (0.01)	12.4 (0.009)	12.8 (0.009)	
*Family poverty level*, *%* (*SE*)					
<100	11.9 (0.007)	20.9 (0.02)	11.5 (0.007)	6.3 (0.007)	.127
100–199	19.0 (0.008)	25.6 (0.01)	19.5 (0.008)	13.8 (0.01)	
200–399	28.8 (0.01)	31.2 (0.01)	30.3 (0.01)	25.6 (0.02)	
≥400	40.2 (0.02)	22.2 (0.02)	38.7 (0.02)	54.3 (0.02)	
Acculturation score, mean (SE)	2.7 (0.02)	2.8 (0.02)	2.7 (0.02)	2.7 (0.03)	.236
*Education*, *%* (*SE*)					
<High school	15.3 (0.009)	23.7 (0.01)	15.9 (0.009)	7.7 (0.008)	.672
High school graduate	20.8 (0.008)	29.6 (0.02)	22.9 (0.01)	12.8 (0.008)	
College degree or more	63.8 (0.01)	46.7 (0.02)	61.1 (0.01)	79.6 (0.01)	
*Frequency of interproximal hygiene*, *%* (*SE*)					
0 days/week	27.7 (0.007)	41.7 (0.01)	28.4 (0.008)	18.4 (0.009)	.129
1–6 days/week	39.4 (0.007)	34.6 (0.01)	39.9 (0.009)	41.5 (0.01)	
7 days/week	32.9 (0.006)	23.7 (0.01)	31.7 (0.009)	40.1 (0.01)	
Comorbidity score, mean (SE)	0.9 (0.01)	0.9 (0.03)	0.9 (0.02)	0.7 (0.02)	.226
BMI, mean (SE)	29.1 (0.1)	29.8 (0.3)	29.8 (0.1)	27.6 (0.2)	.052
*Smoking*, *%* (*SE*)					
Yes	17.4 (0.005)	66.7 (0.01)	11.8 (0.006)	1.0 (0.003)	.349
No or former	82.6 (0.005)	33.3 (0.01)	88.2 (0.006)	98.9 (0.003)	
*Alcohol intake, % (SE)*					
Below recommended limitations	56.1 (0.008)	10.9 (0.009)	52.2 (0.009)	86.9 (0.009)	.672
Above recommended limitations	43.9 (0.008)	89.1 (0.009)	47.8 (0.009)	13.2 (0.009)	
*LTPA*, *%* (*SE*)					
Low LTPA	62.5 (0.009)	92.5 (0.009)	71.9 (0.008)	28.1 (0.01)	.349
High LTPA	37.5 (0.009)	7.5 (0.009)	28.0 (0.008)	71.9 (0.01)	
*HEI score*, *%* (*SE*)					
Unhealthy diet	57.1 (0.009)	93.7 (0.008)	69.5 (0.009)	19.8 (0.01)	.621
Healthy diet	42.9 (0.009)	6.4 (0.008)	30.5 (0.009)	80.2 (0.01)	
*Sleep duration*, *%* (*SE*)					
Insufficient/excessive	39.4 (0.007)	79.4 (0.01)	43.2 (0.009)	11.3 (0.007)	.239
Proper	60.6 (0.007)	20.6 (0.01)	56.8 (0.009)	88.8 (0.007)	
*Periodontitis*, *%* (*SE*)					
Mild	4.3 (0.004)	4.3 (0.006)	4.9 (0.005)	3.3 (0.004)	.*036* *
Moderate	30.1 (0.01)	39.5 (0.02)	29.8 (0.01)	25.7 (0.02)	
Severe	7.8 (0.005)	15.9 (0.01)	7.5 (0.005)	4.1 (0.005)	
WBC (1000 cells/μL), mean (SE)	7.1 (0.04)	7.9 (0.09)	7.1 (0.05)	6.6 (0.05)	.*041* *
C‐reactive protein (mg/dL), mean (SE)	0.4 (0.02)	0.5 (0.08)	0.4 (0.02)	0.3 (0.02)	.*039* *
Segmented neutrophils number (1000 cells/μL), mean (SE)	4.2 (0.03)	4.8 (0.07)	4.2 (0.04)	3.9 (0.04)	.*029* *

Abbreviations: %, percentage; μL, microliters; BMI, body mass index; HEI, healthy eating index; LTPA, leisure‐time physical activity; SE, standard error; WBC, white blood cell count.

* *p* < .05.

** *p* < .01.

*** *p* < .001.

**TABLE 2:** Characteristics of the study population in the UK Biobank cohort.

Variables	Overall (*N* = 111 679)	Healthy lifestyle score			
		0–1 (*N* = 6968)	2–3 (64538)	4–5 (40173)	*p*‐Value
Age (years), mean (SD)	55.7 (7.9)	54.5 (7.6)	55.3 (7.9)	56.7 (7.9)	.067
*Gender*, *n* (*%*)					
Male	45 922 (41.1)	3734 (53.6)	28 568 (44.2)	13 620 (34.0)	.079
Female	65 757 (58.9)	3228 (46.4)	36 112 (55.8)	26 417 (66.0)	
*Ethnicity*, *n* (*%*)					
White	104 521 (93.8)	6784 (97.7)	61 649 (95.4)	36 188 (90.5)	.178
Mixed	592 (0.5)	51 (0.7)	341 (0.5)	200 (0.5)	
Asian/Asian British	3585 (3.3)	47 (0.7)	1307 (2.1)	2231 (5.7)	
Black/Black British	1628 (1.5)	39 (0.6)	818 (1.3)	771 (1.9)	
Other	1019 (0.9)	19 (0.3)	479 (0.8)	521 (1.3)	
*Total household income*, *n* (*%*)					
Less than £18 000	16 774 (17.0)	840 (12.9)	9065 (15.7)	6869 (20.1)	.489
£18 000 to £30 999	22 846 (23.2)	1364 (21.1)	12 805 (22.1)	8677 (25.4)	
£31 000 to £51 999	27 158 (27.5)	1913 (29.6)	16 164 (27.9)	9081 (26.5)	
£52 000 to £100 000	24 432 (24.8)	1810 (27.9)	15 161 (26.2)	7461 (21.8)	
Greater than £100 000	7413 (7.5)	540 (8.3)	4728 (8.2)	2145 (6.3)	
Acculturation score, mean (SD)	2.9 (0.4)	2.9 (0.3)	2.9 (0.4)	2.8 (0.5)	.967
*Education*, *n* (*%*)					
< High school	10 884 (9.8)	526 (7.6)	5725 (8.9)	4633 (11.7)	.072
High school graduate	51 122 (46.1)	3491 (50.3)	29 857 (46.4)	17 774 (44.8)	
College degree or more	48 942 (44.1)	2927 (42.1)	28 745 (44.7)	17 270 (43.5)	
Comorbidity score, mean (SD)	0.8 (0.9)	0.7 (0.6)	0.6 (0.7)	0.7 (0.6)	.589
BMI, mean (SD)	26.8 (4.7)	27.2 (4.5)	26.9 (4.7)	26.5 (4.7)	.432
*Smoking*, *n* (*%*)					
Yes	22 822 (20.4)	5114 (73.5)	16 463 (25.5)	1245 (3.1)	.698
No or former	89 857 (79.6)	1848 (26.5)	48 217 (74.5)	38 792 (96.9)	
*Alcohol intake*, *n* (*%*)					
Below recommended limitations	73 030 (65.5)	717 (10.3)	34 375 (53.2)	37 938 (94.8)	.023*
Above recommended limitations	38 649 (34.6)	6245 (89.7)	30 305 (46.8)	2099 (5.2)	
*LTPA*, *n* (*%*)					
Low LTPA	28 916 (25.9)	5258 (75.5)	21 917 (33.8)	1741 (4.3)	.019*
High LTPA	82 763 (74.2)	1704 (24.5)	42 763 (66.2)	38 296 (95.7)	
*Diet quality*, *n* (*%*)					
Unhealthy diet	91 741 (82.1)	6843 (98.3)	59 558 (92.1)	25 340 (63.3)	.003**
Healthy diet	19 938 (17.9)	119 (1.7)	5122 (7.9)	14 697 (36.7)	
*Sleep duration*, *n* (*%*)					
Insufficient/excessive	32 381 (29.0)	5108 (73.4)	24 063 (37.3)	3210 (8.1)	.015*
Proper	79 298 (70.9)	1854 (26.6)	40 617 (62.7)	36 827 (91.9)	
*Periodontal diseases*, *n* (*%*)					
Bleeding gums	10 841 (9.7)	753 (10.8)	6527 (10.1)	3561 (8.9)	.004**
Painful gums	2100 (1.9)	148 (2.1)	1233 (1.9)	719 (1.8)	
Loose teeth	1996 (1.79)	163 (2.3)	1136 (1.8)	697 (1.7)	
Any periodontal disease	14 937 (13.4)	1064 (15.3)	8896 (13.7)	4977 (12.4)	
WBC (1000 cells/μL), mean (SD)	6.7 (2.2)	7.0 (1.9)	6.7 (1.9)	6.7 (2.7)	.048*
C‐reactive protein (mg/dL), mean (SD)	2.3 (4.1)	2.5 (4.1)	2.4 (4.1)	2.3 (4.0)	.052
Segmented neutrophils number (1000 cells/μL), mean (SD)	4.1 (1.4)	4.3 (1.4)	4.1 (1.3)	4.1 (1.4)	.078

Abbreviations: %, percentage; BMI, body mass index; LTPA, leisure‐time physical activity; SE, standard error; μL, microliters; WBC, white blood cell count.

* *p* < .05.

** *p* < .01.

*** *p* < .001.

In the NHANES, simple and multiple regression analyses confirmed that the presence of 2–3 healthy behaviours (vs. 0–1) was associated with lower odds of periodontitis (OR = 0.5) and severe periodontitis (OR = 0.5). The same direction of association was confirmed when considering linear surrogate measures of periodontal severity. In the UKB, simple and multiple regression analyses identified the presence of 2–3 healthy behaviours (vs. 0–1) was associated with lower odds of bleeding gums (OR = 0.8), painful gums (OR = 0.8), and loose teeth (OR = 0.6). This association increased when considering 4–5 healthy behaviours (vs. 0–1) as exposure in both the NHANES (periodontitis: OR = 0.3; severe periodontitis: OR = 0.1) and the UKB (bleeding gums: OR = 0.9; loose teeth: OR = 0.6), hence indicating the presence of a dose–response relationship between the number of healthy behaviours and periodontal diseases (Table 3). Additional analyses confirmed the same association when stratifying data by age and sex, and when considering each healthy lifestyle independently as exposure (i.e., no smoking, alcohol below the recommended limitations, high LTPA, high diet quality, and ideal sleep duration) (Tables S2–S8). Moreover, sensitivity analyses confirmed the same direction of association in systemically healthy participants (Tables S9 and S10), as well as across subgroups of BMI (Tables S11 and S12), and according to last dental visit, and frequency of interproximal brushing (in NHANES only; Tables S13 and S14).

**TABLE 3:** Association between categories of healthy lifestyle score and periodontal diseases.

Outcomes	Healthy lifestyle score as exposure – OR/MD (95% CI)			
	2–3 healthy lifestyles (vs. 0–1 healthy lifestyles)		4–5 healthy lifestyles (vs. 0–1 healthy lifestyles)	
	Crude	Adjusted	Crude	Adjusted
NHANES				
Periodontitis	*0*.*5* (*0*.*4*, *0*.*6*)***	*0*.*5* ^a^ (*0*.*4*, *0*.*6*)***	*0*.*3* (*0*.*2*, *0*.*4*)***	*0*.*3* ^a^ (*0*.*2*, *0*.*4*)***
*Periodontitis severity*				
Mild periodontitis	*0*.*5* (*0*.*3*, *0*.*6*)***	0.8^b^ (0.5, 1.1)	0.5 (0.3, 0.6)	*0*.*6* ^b^ (*0*.*4*, *0*.*9*)*
Moderate periodontitis	*0*.*4* (*0*.*3*, *0*.*5*)***	*0*.*6* ^b^ (*0*.*4*, *0*.*9*)*	0.4 (0.3, 0.5)	*0*.*3* ^b^ (*0*.*3*, *0*.*4*)***
Severe periodontitis	*0*.*2* (*0*.*1*, *0*.*2*)***	*0*.*5* ^b^ (*0*.*3*, *0*.*8*)**	*0*.*1* (*0*.*1*, *0*.*2*)***	*0*.*1* ^b^ (*0*.*01*, *0*.*2*)***
*PPD*				
*% sites PPD ≥4 mm*	*−2*.*0* (*−2*.*6*, *−1*.*5*)***	*−1*.*3* ^c^ (*−1*.*9*, *−0*.*8*)***	*−3*.*1* (*−3*.*7*, *−2*.*6*)***	*−1*.*9* ^c^ (*−2*.*5*, *−1*.*4*)***
*% sites PPD ≥5 mm*	*−0*.*7* (*−0*.*9*, *−0*.*4*)***	*−0*.*4* ^b^ (*−0*.*7*, *−0*.*2*)**	*−1*.*0* (*−1*.*3*, *−0*.*7*)***	*−0*.*6* ^b^ (*−0*.*9*, *−0*.*4*)***
*% sites PPD ≥6 mm*	*−0*.*2* (*−0*.*4*, *−0*.*09*)**	*−0*.*1* ^c^ (*−0*.*3*, *−0*.*02*)*	*−0*.*3* (*−0*.*5*, *−0*.*2*)***	*−0*.*2* ^c^ (*−0*.*3*, *−0*.*09*)**
*CAL*				
% sites CAL ≥3 mm	*−6*.*6* (*−7*.*6*, *−5*.*6*)***	*−5*.*9* ^d^ (*−6*.*9*, *−4*.*9*)***	*−9*.*6* (*−10*.*7*, *−8*.*5*)***	*−8*.*0* ^d^ (*−9*.*3*, *−6*.*8*)***
% sites CAL ≥4 mm	*−4*.*5* (*−5*.*3*, *−3*.*7*)***	*−4*.*1* ^d^ (*−4*.*9*, *−3*.*3*)***	*−6*.*4* (*−7*.*2*, *−5*.*5*)***	*−5*.*5* ^d^ (*−6*.*4*, *−4*.*5*)***
% sites CAL ≥5 mm	*−2*.*6* (*−3*.*1*, *−2*.*0*)***	*−2*.*3* ^d^ (*−2*.*9*, *−1*.*7*)***	*−3*.*7* (*−4*.*4*, *−3*.*1*)***	*−3*.*1* ^d^ (*−3*.*8*, *−2*.*5*)***
% sites CAL ≥6 mm	*−1*.*5* (*−1*.*9*, *−1*.*1*)***	*−1*.*3* ^e^ (*−1*.*8*, *−0*.*9*)***	*−2*.*3* (*−2*.*7*, *−1*.*8*)***	*−1*.*8* ^e^ (*−2*.*3*, *−1*.*4*)***
*UK Biobank*				
Bleeding gums (vs. healthy)	0.9 (0.8, 1.0)	0.9^f^ (0.8, 1.0)	*0*.*8* (*0*.*7*, *0*.*9*)***	*0*.*8* ^f^ (*0*.*7*, *0*.*9*)***
Painful gums (vs. healthy)	0.9 (0.7, 1.1)	0.9^g^ (0.7, 1.0)	0.9 (0.8, 1.1)	*0*.*7* ^g^ (*0*.*6*, *0*.*9*)***
Loose teeth (vs. healthy)	*0*.*7* (*0*.*6*, *0*.*8*)***	*0*.*6* ^f^ (*0*.*5*, *0*.*7*)***	*0*.*6* (*0*.*5*, *0*.*7*)***	*0*.*5* ^f^ (*0*.*4*, *0*.*6*)***
Any periodontal disease (vs. healthy)	*0*.*8* (*0*.*8*, *0*.*9*)**	*0*.*8* ^f^ (*0*.*7*, *0*.*9*)***	*0*.*8* (*0*.*7*, *0*.*9*)***	*0*.*8* ^f^ (*0*.*7*, *0*.*9*)***

*Note*: The following confounders were tested using univariate analysis in order to obtain the adjusted estimates: age, gender, ethnicity, income, acculturation score, education, body mass index, frequency of self‐performed oral hygiene (NHANES), last dental visit (NHANES), number of comorbidities (comorbidity score).

Abbreviations: %, percentage; CAL, clinical attachment level; CI, confidence interval; MD, difference in means; mm, millimetre; OR, odds ratio; PPD, probing pocket depth.

^a^ Adjusted for age, gender, and body mass index.

^b^ Adjusted for age, body mass index, income, education.

^c^ Adjusted for age, gender, income, comorbidity score.

^d^ Adjusted for age, acculturation score, education, comorbidity score.

^e^ Adjusted for age, gender, body mass index, education, frequency of self‐performed oral hygiene (NHANES).

^f^ Adjusted for age, ethnicity, body mass index, education.

^g^ Adjusted for age, gender, body mass index, education.

* *p* < .05.

** *p* < .01.

*** *p* < .001.

Furthermore, mediation analyses highlighted how the association between the presence of ≥2 healthy behaviours and periodontal diseases was mildly explained by markers of systemic inflammation, with a proportion of mediated effect ranging between 2% and 6% for NHANES, and between 1% and 14% for UKB (Table 4). All markers of systemic inflammation correlated with the prevalence of periodontal diseases and surrogate measures (Table S15).

**TABLE 4:** Mediation analyses for the association between HL score >1 and periodontitis/severe periodontitis.

	WBC count			CRP			Neutrophils count			
	Effect estimate/Coefficient	SE	*p*‐Value	Effect estimate/Coefficient	SE	*p*‐Value	Effect estimate/Coefficient	SE		*p*‐Value
*NHANES*										
Periodontitis										
a (exposure → mediator)	−0.232	0.078	<.*001* ***	−0.172	0.04	<.*001* ***	−0.411	0.030		<.*001* ***
b (mediator → outcome)	0.010	0.003	.*001* **	0.027	0.02	.118	0.010	0.002		<.*001* ***
c (total effect)	−0.224	0.012	.*001* **	−0.183	0.03	.*001* **	−0.110	0.003		.*001* **
c’ (direct effect)	−0.211	0.02	<.*001* ***	−0.178	0.03	<.*001* ***	−0.105	0.008		<.*001* ***
ab (mediated effect)	−0.012	0.004	.*001* **	−0.005	0.003	.142	−0.004	0.001		<.*001* ***
ab/c (percentage mediated) = %	5.5	–	–	2.5	–	–	4.0	–		–
Propensity scores for the matched sample	−0.451	0.013	<.*001* ***	−0.421	0.026	<.*001* ***	−0.278	0.017		<.*001* ***
Severe periodontitis										
a (exposure → mediator)	−0.554	0.504	<.*001* ***	−0.063	0.024	.*001* **	−0.375	0.042		<.*001* ***
b (mediator → outcome)	0.012	0.002	<.*001* ***	0.043	0.014	.*001* **	0.013	0.002		<.*001* ***
c (total effect)	−0.107	0.002	<.*001* ***	−0.110	0.002	<.*001* ***	−0.106	0.001		<.*001* ***
c’ (direct effect)	−0.100	0.007	<.*001* ***	−0.107	0.014	<.*001* ***	−0.102	0.008		<.*001* ***
ab (mediated effect)	−0.006	0.001	<.*001* ***	−0.003	0.001	.*043* *	−0.005	0.001		<.*001* ***
ab/c (percentage mediated) = %	6.0	–	–	2.0	–	–	5.0	–		–
Propensity scores for the matched sample	−0.284	0.036	<.*001* ***	−0.271	0.029	<.*001* ***	−0.296	0.031		<.*001* ***
*UK Biobank*										
Bleeding gums										
a (exposure → mediator)	−0.127	0.010	<.*001* ***	−0.086	0.022	.*001* **	−0.077	0.007	<.*001* ***	
b (mediator → outcome)	0.003	0.002	<.*001* ***	0.001	0.0002	<.*001* ***	0.003	0.001	<.*001* ***	
c (total effect)	−0.014	0.001	.*001* **	−0.013	0.0001	.*001* **	−0.014	0.002	<.*001* ***	
c’ (direct effect)	−0.013	0.002	<.*001* ***	−0.014	0.002	<.*001* ***	−0.013	0.004	<.*001* ***	
ab (mediated effect)	−0.001	0.001	<.*001* ***	−0.001	0.0001	<.*001* ***	−0.001	0.0001	<.*001* ***	
ab/c (percentage mediated) = %	3.0	–	–	1.0	–	–	2.0	–	–	
Propensity scores for the matched sample	−0.015	0.002	<.*001* ***	−0.016	0.002	<.*001* ***	−0.017	0.003	<.*001* ***	
Loose teeth										
a (exposure → mediator)	−0.151	0.010	<.*001* ***	−0.092	0.023	<.*001* ***	−0.093	0.008		<.*001* ***
b (mediator → outcome)	0.006	0.0003	<.*001* ***	0.002	0.0002	<.*001* ***	0.007	0.0004		<.*001* ***
c (total effect)	0.007	0.001	<.*001* ***	0.003	0.001	<.*001* ***	0.002	0.0002		<.*001* ***
c’ (direct effect)	−0.006	0.003	.096	−0.007	0.002	.*010* *	−0.006	0.002		<.*001* ***
ab (mediated effect)	−0.001	0.0001	<.*001* ***	−0.001	0.001	<.*001* ***	−0.001	0.0001		<.*001* ***
ab/c (percentage mediated) = %	14.0	–	–	2.0	–	–	10.0	–		–
Propensity scores for the matched sample	−0.0009	0.001	.*006* **	−0.002	0.006	.*016* *	−0.0009	0.001		.057

Abbreviations: CI, confidence interval; CRP, C‐reactive protein; HL, healthy lifestyle; SE, standard error; WBC, white blood cell count.

* *p* < .05.

** *p* < .01.

*** *p* < .001.

## 4 DISCUSSION

The findings from this study indicate that the presence of at least 2 healthy lifestyle behaviours is associated with lower prevalence of any periodontal diseases. Furthermore, the association between healthy lifestyles and periodontal diseases exhibited a potential dose–response relationship, with a higher number of healthy behaviours being associated with lower odds of periodontal diseases. Limited evidence of a potential mediation of this association based on systemic inflammation (as assessed by leucocyte counts) was noted.

Despite the limited evidence available on the association between healthy lifestyles in their entirety and oral health, the results obtained in the current study are consistent with those achieved in previous prospective investigations.^29^, ^30^ Iwasaki and coworkers (2018) demonstrated a protective effect of the combined adherence to multiple healthy lifestyles (non‐smoking, physical activity, healthy body weight, and high diet quality) when assessing the onset of periodontitis and tooth loss over a period of 6 years in a population of older Japanese adults. Experimental evidence from a prospective investigation highlighted the possible impact of lifestyle overall indicators on the response to the treatment of periodontitis. Indeed, participants exposed to either unhealthy diet, physical inactivity, high stress, and poor sleep quality struggled to achieve the expected clinical endpoints after 3 months of completion of management of periodontitis.^30^ Moreover, prevalence estimates of periodontitis and periodontal diseases in the NHANES and UKB databases (42.2% and 13.4%, respectively) are consistent with previous investigations using the same population samples (42.0% and 14.8%, respectively).^5^, ^31^

Possible biological mechanisms underpinning a protective effect of healthy lifestyles on the periodontium are still poorly described and understood. The available evidence regarding the local and systemic repercussions of each individual lifestyle factor points towards shared mechanisms of modulation of inflammation including oxidative stress production^32^, ^33^ and their impact on the risk of periodontitis.^34^, ^35^ A bi‐directional link between periodontitis and systemic inflammation is further corroborated in this study by the potential mediation‐effect of inflammatory biomarkers on the prevalence of periodontal diseases.

These results are in line with the current evidence focusing on lifestyle as a combination of behaviours instead of considering each of them separately. Indeed, several observational studies demonstrated that a combination of healthy lifestyles is associated with lower mortality, incident cardiovascular disease and diabetes.^22^, ^36^

As lifestyles behaviours/factors are closely intertwined, it could be hypothesized that individuals with patterns of unhealthy behaviours are less likely to follow domiciliary oral hygiene instructions and vice versa.^17^, ^37^ Furthermore, it has already been suggested that unhealthy lifestyles such as high stress or the consumption of highly palatable foods are associated with higher dental plaque accumulation.^17^, ^38^ Given the recent confirmation of the importance of oral health for achieving good general health, promotion of healthy lifestyle could be revisited to also include better oral health behaviours. Indeed, a more comprehensive approach in promoting better lifestyles could help reducing the burden of NCDs and multimorbidities as they share most common risk factors and this could explain the associations found in this study. A common risk‐factor approach is particularly important given the increasing prevalence of oral diseases and NCDs over the life course in the world's ageing population.^39^, ^40^

It is noteworthy to mention that these analyses present some limitations including the risk of residual confounding and the cross‐sectional design of the data obtained, which limit our ability to assess causality of the associations found, the impact of the duration of healthy lifestyle behaviours on periodontal health, as well as the validity of the mediation analysis. Future well‐designed cohort studies should evaluate the impact of adopting healthy lifestyles on the incidence of future periodontal diseases. Information bias cannot be excluded in these analyses because of the use of self‐reported assessment of lifestyles in both populations, as well as of the periodontal status in the UKB. In particular, recent evidence demonstrated good diagnostic accuracy for periodontal diseases definitions for self‐reported measures of painful gums and loose teeth, but not for bleeding gums.^41^ This could have affected the estimates obtained from the analyses. Along the same line, while in the NHANES dataset, it was possible to use a recognized case definition, in the UKB self‐reported measures of periodontal diseases were available. As not all components of a healthy lifestyle were measured (i.e., stress levels), we urge caution in interpreting the results as the composite variable of a healthy lifestyle could not account for every different lifestyles' combination or for their magnitude of effect on periodontal and general health. Lastly, although the direction of association between HLS and periodontal diseases was similar across two different datasets (US and UK populations), it should be noted that we cannot exclude measurement bias with regard to both the exposure and the outcome; hence, caution should be paid when generalizing the study results. On the other hand, this is the first comprehensive analysis of the potential impact of healthy lifestyle behaviours on periodontal tissues using two large surveys of the Western world.

However, this is the first comprehensive analysis to assess a potential impact of healthy lifestyle behaviours on the periodontium using two large representative surveys.

## 5 CONCLUSIONS

Healthy lifestyle behaviours are associated with lower prevalence of periodontal diseases in both populations in a potential dose–response manner, and they are partially mediated by systemic inflammation. Furthermore, studies should investigate the effects of promoting healthy lifestyles to improve both oral and general health.

## AUTHOR CONTRIBUTIONS

CM and FD designed the study, conducted the data analysis, and drafted the manuscript. SL, SBH, and JS critically revised the manuscript for important intellectual content. All authors approved the final version of the manuscript. FD is guarantor. The corresponding author attests that all the listed authors meet authorship criteria and that no others meeting the criteria have been omitted.

## CONFLICT OF INTEREST STATEMENT

The authors received no financial support, and they declare no potential conflicts of interest with respect to the authorship and/or publication of this article.

## Supporting information


Table S1.

## Data Availability

NHANES data are publicly available at www.cdc.gov/nchs/nhis/index.htm, and data from UK Biobank are available upon application at www.ukbiobank.ac.uk/register‐apply.
