# Supplementary material for: Healthy lifestyles and better periodontal health: Results from two large population‐based surveys
Source: J Periodontal Res. 2024 Jul 2;60(2):144–53. doi: 10.1111/jre.13320 (PMC11873680; doi:10.1111/jre.13320)
Supplement: Supplementary file 1 — Table S1. [file JRE-60-144-s001.docx]

**Appendix 1.**  Exposure variables’ assessment methods (lifestyles).

**NHANES**

- Cigarette smoking (self-reported)*: non-smokers/smokers -* subjects who reported currently smoking every day or some days - SMQ040 - and those who had smoked at least 100 cigarettes during life - SMQ020 - were considered as current smokers;
- Alcohol intake (ALQ130, ALQ101; self-reported): identified considering the recommended alcohol limitations as threshold (ALQ130; 14 g/d or 1 standard alcoholic drink /d for women, and 28 g/d or 2 standard alcoholic drinks /d for men). Participants were categorized as being *below* (if they had not drunk at least 12 alcohol drinks/1 year - ALQ101, or if they had drunk at least 12 alcohol drinks/1 year but they were below the recommended limitations) or *above* (if they had drunk at least 12 alcohol drinks/1 year and they were above the recommended limitations) the recommended alcohol limitations (Ricci et al., 2020);
- Physical activity: moderate-to-vigorous intensity leisure-time physical activity (MVPA) was assessed using the Global Physical Activity Questionnaire (GPAQ) (PAQ650; PAQ655; PAD660; PAQ665; PAQ670; PAD675). Metabolic Equivalent Times (MET min/week) were calculated for each participant by adding the time spent on each activity weighted by its metabolic equivalent score. The assumed MET scores for MVPA are 4.0 for moderate intensity PA, and 8.0 for vigorous intensity PA (Janssen et al., 2013). According to previous studies, participants were categorized as being *active (vs. inactive)* if they are in the top third of the total leisure-time physical activity level (in terms of MET min/week) as previously reported (Abdullah Said et al., 2018; Janssen et al., 2013; Y. Zhang et al., 2021);
- Diet quality: the dietary intake data were obtained from two 24-h recall interviews, which were conducted by the trained personnel using an automated multiple-pass method. The first interview was arranged face-to-face at the Mobile Examination Center (MEC) whilst the second was carried out over the phone 3–10 days later. The energy value and nutrients for each food or beverage intake were calculated using the Food and Nutrient Database for Dietary Studies (FNDDS) and grouped using the Food Patterns Equivalence Database (FPED) from the US Department of Agriculture (USDA). The diet quality was estimated using the Healthy Eating Index (HEI-2015). This contains 13 components (food groups or nutrients), including 9 adequacy components (total vegetables, greens and beans, total fruits, whole fruits, whole grains, dairy, total protein foods, seafood and plant proteins and fatty acids) and 4 moderation components (sodium, refined grains, saturated fats and added sugars). All components were expressed as amounts per 1000 kcal except for fatty acids (expressed as a ratio of unsaturated to saturated fats), saturated fats (expressed as % energy) and added sugars (expressed as % energy). These components were scored separately and incorporated into a total score up to 100. For each participant, the final dietary intake was estimated using the mean value of the two 24hrs recall data. The variables collected during the first (https://wwwn.cdc.gov/Nchs/Nhanes/2009-2010/DR1TOT_F.htm) and the second (https://wwwn.cdc.gov/Nchs/Nhanes/2009-2010/DR2TOT_F.htm) day of interview were used to compute the HEI-2015. For each survey cycle, the FPED were downloaded from the USDA website (<https://www.ars.usda.gov/northeast-area/beltsville-md-bhnrc/beltsville-human-nutrition-research-center/food-surveys-research-group/docs/fped-databases/>). Participants were defined as having a healthy (top 2 quintiles) or unhealthy (bottom three quintiles) diet based on the distribution of the HEI-2015.
- Sleep duration: According to the National Sleep Foundation guidelines, participants were categorized as having a *proper sleep duration* (SLD010H) if they were adults (30-64 years) with 7-9 hours of sleep duration, or if they were elderlies (≥65 years) with 7-8 hours of sleep duration, otherwise they were categorized as having an excessively *short/long sleep duration* (Hirshkowitz et al., 2015).

**UKB**

- Cigarette smoking (22506, 2644; self-reported)*: non-smokers/smokers-* subjects who reported currently smoking on most or all days, or occasionally - 22506 - and those who had smoked at least 100 cigarettes during life - 2644 - were considered as current smokers (Zhang et al., 2021);
- Alcohol intake (1558; self-reported): Participants were asked about the frequency of drinking alcohol (1558), i.e., (almost) daily, three or four times a week, once or twice a week, one to three times a month, special occasions only, never, and prefer not to answer. Participants were then categorized as drinking more or less than the recommended limitations (Ricci et al., 2020);
- Physical activity (104900, 104910, 104920, 11005, 11006, 884, 894, 904, 914, 3637, 3647; self-reported): Level of leisure-time physical activity in the UKB was assessed based on three types of activities; walking for pleasure (assigned 3.3 MET equivalents), strenuous sports (assigned 8 MET equivalents), and other exercises (i.e., swimming, cycling, keep fit, bowling) (assigned 4 MET equivalents). The frequency and duration for each time was asked for each participant. Participants could choose one of the following frequency options, i.e., once in the last 4 weeks, 2-3 times in the last 4 weeks, once a week, 2-3 times a week, 4-5 times a week, every day, do not know, and prefer not to answer. Options including a range would be substituted by the midpoint of the range, e.g., we assigned 2.5 times a week for the option “2-3 times a week”. The duration for each time was also obtained by some options, i.e., less than 15 minutes, between 15 and 30 minutes, between 30 minutes and 1 hour, between 1 and 1.5 hours, between 1.5 and 2 hours, between 2 and 3 hours, over 3 hours, do not know, and prefer not to answer. Each option would be substituted by the midpoint of the range, and those who chose over 3 hours were substituted by 3 hours as recommended by other studies. Participants were categorized as being active (vs. inactive) if they were in the top third of the total leisure-time physical activity level (in terms of MET min/week), otherwise they were defined as inactive as previously reported (Abdullah Said et al., 2018; Janssen et al., 2013; Zhang et al., 2021).
- Diet quality: Dietary quality was evaluated as ideal or poor as previously reported. Ideal diet was defined as having an adequate intake of at least half of the following dietary components: increased consumption of fruits, vegetables, whole grains, (shell)fish, dairy products, and vegetable oils; and reduced or no consumption of refined grains, (un)processed meats and sugar-sweetened beverages. Fruit intakes were evaluated according to daily consumptions of fresh fruit (pieces) and dried fruit (pieces). Vegetable intakes were evaluated according to daily consumptions of cooked vegetables (tablespoons) and salad/raw vegetables (tablespoons). Wholegrain intakes were evaluated according to weekly consumptions of bread (slices, if the participant consumed whole meal/whole grain bread) and cereal (bowls, if the participant consumed bran, oat, or Muesli). (Shell)fish were evaluated according to the frequency of eating oily fish and non-oily fish. Dairy intakes were evaluated according to the frequency of cheese consumption and whether the participant consumed milk. Vegetable oils were evaluated according to the weekly consumption of bread (slices) if the participants consumed flora pro-active/benecol, soft margarine, olive oil based, polyunsaturated/sunflower oil based, or other low/reduced fat spread. Refined grain intakes were evaluated according to weekly consumptions of bread (slices, if the participant consumed white, brown, or other bread) and cereal (bowls, if the participant consumed biscuit or others). Processed meat consumptions were evaluated according to the frequency of consumption and whether the participants did not eat it anymore (according to a question about age when last ate meat). Unprocessed meat consumptions were evaluated according to the frequency of consumption of poultry, beef, lamb/mutton, and pork and whether the participants did not eat meat anymore (according to a question about age when last ate meat). Sugar-sweetened beverage intakes were evaluated by a question “Which of the following do you NEVER eat?” Those who chose sugar or foods/drinks containing sugar were regarded as never drinking sugar-sweetened beverages. The diet components, the intake goal, the field IDs, and the amount per serving considered were calculated using the same score as previously reported (Zhang et al., 2021). Participants were categorized as having a healthy diet if they had the ideal intake of 5 or more dietary components, otherwise they were considered as having an unhealthy diet.
- Sleep duration: According to the National Sleep Foundation guidelines, participants were categorized as having a *proper sleep duration* (1160) if they were adults (30-64 years) with 7-9 hours sleep duration, or if they were elderlies (≥65 years) with 7-8 hours sleep duration, otherwise they were categorized as having an excessively *short/long sleep duration* (Hirshkowitz et al., 2015).

**HEALTHY LIFESTYLE SCORE**

For each lifestyle factor, the healthy level was assigned 1 point, while the unhealthy level was assigned 0 points. Thus, the healthy lifestyle score was the sum of the points, and ranges between 0 and 5, with higher scores indicating healthier lifestyles. This additive method had been widely used in the medical literature (Zhang et al., 2020; Zhang et al., 2021; Zhang et al., 2021), but it rests on the assumption that the associations between the different lifestyle factors and the outcomes are identical, which might not be true. Therefore, all the individual estimates of association between the lifestyle factors and periodontitis were also computed (see Supplementary Tables).

**Appendix 2.**  Confounders’ assessment methods and categories boundaries.

**NHANES**

The following set of confounders was tested:

- Age (RIDAGEYR; *self-reported*);

- Sex (RIAGENDR; *self-reported; male/female*);

- Ethnicity (RIDRETH1/RIDRETH3; *Mexican American, non-Hispanic white, non-Hispanic black, other race*);

- Family Poverty Level (FPL) (INDFMPIR; self-reported; *<100%, 100-199%, 200-399%, ≥400%*);

- Acculturation score: A 3-point acculturation score was constructed according to the country of birth (DMDBORN2/ DMDBORN4) and length of time in the US (DMDYRSUS). A 3-point score was assigned to the country of birth and length of time in the US, i.e., 3 points for US-born, 2 points for foreign-born and lived in the US ≥20 years, 1 point for foreign-born and lived in the US 10 to 19 years, and 0 points for foreign-born and lived in the US <10 years. The higher the score, the more acculturated the participants (Y. B. o. Zhang et al., 2021);

- Educational level (DMDEDUC2*; self-reported; <high school, high school, >high school*);

- Body Mass Index (BMI; BMXBMI*; the following formula was used “weight/height^2^”;* weight was measured with a digital scale in kilograms, while height was measured with a portal stadiometer in meters;

- Frequency of self-performed interproximal oral hygiene (OHQ870; *0 days/week, 1-6 days/week, 7 days/week*);

- Last dental visit (OHQ030*; more or less than 6 months*)

- Comorbidity score: a 4-point score was built to account for the number of comorbidities. The following comorbidities were identified: i) diabetes (*no diabetes* - glycated hemoglobin (HbA1c; LBXGH) <6.5%, and serum glucose at 2 hours following a 75 g glucose load (OGTT; LBXGLT) <200 mg/dL, and fasting plasma glucose (FPG; LBXGLU) <126 mg/dL, and no self-reported diagnosis of diabetes - DIQ010, and *diabetes* - HbA1c- ≥6.5%, or OGTT ≥200 mg/dL, or FPG ≥126 mg/dL, or self-reported diagnosis of diabetes); ii) hypertension (*no hypertension* - measured Systolic Blood Pressure (SBP) < 120 mmHg and measured Diastolic Blood Pressure (DBP) < 80mmHg, *borderline hypertension* - 120≤SBP<140 mmHg or 80≤DBP<90 mmHg, *hypertension* - average of SBP≥140mmHg or DBP≥90mmHg); iii) cardiovascular or cerebrovascular diseases (*yes* - if participant reported history of congestive heart failure - MCQ160b, or coronary heart disease - MCQ160c, or angina/angina pectoris - MCQ160d, or heart attack - MCQ160e, or stroke - MCQ160f, and *no* if otherwise); iv) depressive symptoms (DPQ010/ DPQ020/ DPQ030/ DPQ040/ DPQ050/ DPQ060/ DPQ070/ DPQ080 / DPQ090; assessed with the Patient Health Questionnaire - PHP-9; participants had depressive symptoms whenever PHP-9 ≥ 10) (Manea et al., 2012).

**UKB**

The following set of confounders was tested:

- Age (21003; *self-reported*), defined as the “Age when attended the assessment center”;

- Sex (31; *self-reported; male/female*);

- Ethnicity (21000; *white, mixed, Asian/Asian British, Black/Black British*);

- Total household income before tax (738; self-reported; “*less than ₤18 000”/“₤18 000 to 30 999”/ “₤31 000 to 51 999”/“₤52 000 to 100 000”/“greater than ₤100 000”).*

- Acculturation score: A 3-point acculturation score was constructed according to the country of birth (1647; *UK/elsewhere*) and length of time in the UK (3659; *“Year immigrated to the UK”*). A 3-point score was assigned to the country of birth and length of time in the UK, i.e., 3 points for UK-born, 2 points for foreign-born and lived in the UK ≥20 years, 1 point for foreign-born and lived in the UK 10 to 19 years, and 0 points for foreign-born and lived in the UK <10 years. The higher the score, the more acculturated the participants (Y. B. o. Zhang et al., 2021);

- Education attainment (6138*; “College or university degree”/“A levels/AS levels or equivalent”/“O levels/GCSEs or equivalent”/“CSEs or equivalent”/“NVQ or HND or HNC or equivalent”/“Other professional qualifications”/“None of the above” (equivalent to less than high school diploma)/“Prefer not to answer*);

- Body Mass Index (BMI; 21001*; the following formula was used “weight/height^2^*”): weight was measured with a digital scale in kilograms, while height was measured with a portal stadiometer in meters;

- Comorbidity score: a 4-point score was built to account for the number of comorbidities. The following comorbidities were identified: i) diabetes (2443; *no diabetes* - glycated hemoglobin (HbA1c; LBXGH) <6.5%, and serum glucose at 2 hours following a 75 g glucose load (OGTT; LBXGLT) <200 mg/dL, and fasting plasma glucose (FPG; LBXGLU) <126 mg/dL, and no self-reported diagnosis of diabetes - DIQ010, and *diabetes* - HbA1c- ≥6.5%, or OGTT ≥200 mg/dL, or FPG ≥126 mg/dL, or self-reported diagnosis of diabetes); ii) hypertension (*no hypertension* - measured Systolic Blood Pressure (SBP) < 120 mmHg and measured Diastolic Blood Pressure (DBP) < 80mmHg, *borderline hypertension* - 120≤SBP<140 mmHg or 80≤DBP<90 mmHg, *hypertension* - average of SBP≥140mmHg or DBP≥90mmHg); iii) cardiovascular or cerebrovascular diseases (131059/131375/131377; *yes/no)*.; iv) bipolar/major depression status (20126).

**Appendix 3.**  Mediators’ assessment methods and categories boundaries.

**NHANES**

Potential mediators included:

- White blood cells count (LBXWBCSI; *1000 cells/uL*);

- C-Reactive Protein (LBXCRP; *mg/dL*);

- Segmented neutrophils number (LBDNENO; *1000 cells/uL*).

**UKB**

Potential mediators included:

- White blood cells count (30000; *1000 cells/uL*);

- C-Reactive Protein (30710; *mg/dL*);

- Neutrophils count (30140; *1000 cells/uL*).

**Table S1**. Missing values for each variable.

|  | **NHANES** | | **UK Biobank** | |
| --- | --- | --- | --- | --- |
| **Variables** | **Collected values – N (%)** | **Missing values – N (%)** | **Collected values – N (%)** | **Missing values – N (%)** |
| Healthy lifestyle score | 9,854 (100.0) | 0 (0.0) | 111,679 (100.0) | 0 (0.0) |
| Periodontitis | 9,854 (100.0) | 0 (0.0) | 111,679 (100.0) | 0 (0.0) |
| Age | 9,854 (100.0) | 0 (0.0) | 111,679 (100.0) | 0 (0.0) |
| Gender | 9,854 (100.0) | 0 (0.0) | 111,679 (100.0) | 0 (0.0) |
| Ethnicity | 9,854 (100.0) | 0 (0.0) | 111,600 (99.9) | 79 (0.1) |
| Family Poverty Level/Total household income | 9,076 (92.1) | 778 (7.9) | 98,773 (88.4) | 13,175 (11.8) |
| Acculturation score | 9,732 (98.8) | 122 (1.2) | 111,600 (99.9) | 79 (0.1) |
| Education | 9,842 (99.9) | 12 (0.1) | 111,174 (99.5) | 505 (0.5) |
| BMI | 9,800 (99.5) | 54 (0.5) | 111,454 (99.5) | 225 (0.5) |
| Frequency of interproximal hygiene | 9,851 (99.07) | 3 (0.03) | - | - |
| Comorbidity score | 8,960 (89.3) | 894 (9.7) | 111,679 (100.0) | 0 (0.0) |
| Smoking | 9,854 (100.0) | 0 (0.0) | 111,679 (100.0) | 0 (0.0) |
| Alcohol intake | 9,854 (100.0) | 0 (0.0) | 111,679 (100.0) | 0 (0.0) |
| LTPA | 9,854 (100.0) | 0 (0.0) | 111,679 (100.0) | 0 (0.0) |
| HEI/Diet quality | 9,854 (100.0) | 0 (0.0) | 111,679 (100.0) | 0 (0.0) |
| Sleep duration | 9,854 (100.0) | 0 (0.0) | 111,679 (100.0) | 0 (0.0) |
| WBC (1000 cells/uL) | 9,602 (97.4) | 252 (2.6) | 107,610 (96.4) | 4,069 (3.6) |
| C-reactive protein (mg/dL) | 3,344 (97.1) | 99 (2.9) | 105,498 (94.5) | 6,181 (5.5) |
| Segmented neutrophils number (1000 cells/uL) | 9,584 (100.0) | 0 (0.0) | 107,384 (96.2) | 4,295 (3.8) |

Abbreviations. BMI, body mass index; HEI, healthy eating index; LTPA, leisure-time physical activity; uL, microliters; WBC, white blood cell count; %, percentage.

**Table S2**. Association between categories of healthy lifestyle score (2-3 vs. 0-1 healthy lifestyles) and periodontitis by subgroups of age and sex.

| **2-3 healthy lifestyles (vs. 0-1 healthy lifestyles) as exposure – OR/MD (95% CI)** | | | | | | | | | | | | | | | | | | |
| --- | --- | --- | --- | --- | --- | --- | --- | --- | --- | --- | --- | --- | --- | --- | --- | --- | --- | --- |
| **Outcomes** | | | **Age** | | | | | | | | **Sex** | | | | | | | |
|  |  |  | **< 60 years** | | | **≥ 60 years** | | | | | **Males** | | | | **Females** | | | |
|  | | | ***Crude*** | ***Adjusted*** | | ***Crude*** | | ***Adjusted*** | | | ***Crude*** | | ***Adjusted*** | | ***Crude*** | | ***Adjusted*** | |
| **NHANES** |  |  | | |  | |  | |  |  | |  | |  | |  | |  |
| **Periodontitis** | | | ***0.4***  ***(0.4, 0.5)^***^*** | ***0.5***  ***(0.4, 0.6)^***^*** | | ***0.5***  ***(0.3, 0.7)^***^*** | | ***0.5***  ***(0.3, 0.7)^**^*** | | | ***0.5***  ***(0.4, 0.6)^***^*** | | ***0.5***  ***(0.4, 0.7)^***^*** | | ***0.5***  ***(0.4, 0.6)^***^*** | | ***0.5***  ***(0.4, 0.6)^***^*** | |
| **Periodontitis severity** | | |  |  | |  | |  | | |  | |  | |  | |  | |
| *Mild periodontitis* | | | ***0.4***  ***(0.3, 0.6)^***^*** | ***0.6***  ***(0.4, 0.9)^***^*** | | 1.7  (0.4, 7.4) | | 1.3  (0.3, 6.0) | | | ***0.4***  ***(0.2, 0.7)^**^*** | | 0.6  (0.3, 1.0) | | ***0.5***  ***(0.3, 0.9)^*^*** | | 0.6  (0.4, 1.0) | |
| *Moderate periodontitis* | | | ***0.3***  ***(0.2, 0.3)^***^*** | ***0.4***  ***(0.3, 0.5)^***^*** | | ***0.4***  ***(0.3, 0.7)^**^*** | | ***0.4***  ***(0.2, 0.8)^**^*** | | | ***0.4***  ***(0.3, 0.5)^***^*** | | ***0.4***  ***(0.3, 0.5)^***^*** | | ***0.5***  ***(0.4, 0.6)^***^*** | | ***0.4***  ***(0.3, 0.6)^***^*** | |
| *Severe periodontitis* | | | ***0.1***  ***(0.08, 0.2) ^***^*** | ***0.2***  ***(0.1, 0.3) ^***^*** | | ***0.1***  ***(0.08, 0.2) ^***^*** | | ***0.2***  ***(0.1, 0.3) ^***^*** | | | ***0.2***  ***(0.1, 0.2) ^***^*** | | ***0.2***  ***(0.1, 0.3) ^***^*** | | ***0.1***  ***(0.09, 0.2) ^***^*** | | ***0.1***  ***(0.08, 0.2) ^***^*** | |
| **PPD** | | |  |  | |  | |  | | |  | |  | |  | |  | |
| % sites PPD ≥ 4 mm | | | ***-1.9***  ***(-2.6, -1.4)^***^*** | ***-1.1***  ***(-1.7, -0.5)^**^*** | | ***-2.3***  ***(-3.7, -0.9)^**^*** | | ***-1.7***  ***(-3.0, -0.4)^*^*** | | | ***-2.1***  ***(-2.9, -1.4)^***^*** | | ***-1.1***  ***(-1.9, -0.2)^*^*** | | ***-1.7***  ***(-2.5, -0.9)^***^*** | | ***-1.3***  ***(-2.1, -0.4)^**^*** | |
| % sites PPD ≥ 5 mm | | | ***-0.6***  ***(-0.9, -0.3)^***^*** | ***-0.3***  ***(-0.6, -0.05)^*^*** | | ***-0.8***  ***(-1.4, -0.1)^*^*** | | -0.6  (-1.1, 0.03) | | | ***-0.7***  ***(-1.1, -0.3)^**^*** | | ***-0.3***  ***(-0.8, 0.1)*** | | ***-0.5***  ***(-0.9, -0.2)^**^*** | | ***-0.4***  ***(-0.8, -0.08)^*^*** | |
| % sites PPD ≥ 6 mm | | | ***-0.2***  ***(-0.4, -0.06)^**^*** | -0.1  (-0.3, 0.04) | | ***-0.2***  ***(-0.4, -0.02)^*^*** | | -0.1  (-0.3, 0.08) | | | ***-0.2***  ***(-0.4, -0.02)^**^*** | | -0.08  (-0.3, 0.1) | | ***-0.2***  ***(-0.3, -0.05)^**^*** | | ***-0.1***  ***(-0.3, -0.08)^*^*** | |
| **CAL** | | |  |  | |  | |  | | |  | |  | |  | |  | |
| % sites CAL ≥ 3 mm | | | ***-7.0***  ***(-8.2, -5.8)^***^*** | ***-5.2***  ***(-6.3, -3.9)^***^*** | | ***-9.5***  ***(-12.8, -6.3)^***^*** | | ***-7.9***  ***(-10.7, -5.2)^***^*** | | | ***-7.4***  ***(-8.9, -5.8)^***^*** | | ***-6.1***  ***(-7.7, -4.4)^***^*** | | ***-5.1***  ***(-6.5, -3.6)^***^*** | | ***-4.8***  ***(-6.3, -3.2)^***^*** | |
| % sites CAL ≥ 4 mm | | | ***-4.6***  ***(-5.5, -3.6)^***^*** | ***-3.6***  ***(-4.6, -2.6)^***^*** | | ***-7.2***  ***(-9.6, -4.9)^***^*** | | ***-5.9***  ***(-7.9, -3.8)^***^*** | | | ***-4.9***  ***(-6.1, -3.8)^***^*** | | ***-4.1***  ***(-5.5, -2.7)^***^*** | | ***-3.4***  ***(-4.6, -2.3)^***^*** | | ***-3.3***  ***(-4.5, -2.1)^***^*** | |
| % sites CAL ≥ 5 mm | | | ***-2.6***  ***(-3.3, -1.9)^***^*** | ***-2.0***  ***(-2.8, -1.3)^***^*** | | ***-4.2***  ***(-6.0, -2.5)^***^*** | | ***-3.2***  ***(-4.8, -1.7)^***^*** | | | ***-2.9***  ***(-3.9, -1.9)^***^*** | | ***-2.3***  ***(-3.3, -1.2)^***^*** | | ***-1.9***  ***(-2.5, -1.2)^***^*** | | ***-1.8***  ***(-2.6, -1.1)^***^*** | |
| % sites CAL ≥ 6 mm | | | ***-1.5***  ***(-2.0, -1.1)^***^*** | ***-1.1***  ***(-1.7, -0.6)^***^*** | | ***-2.5***  ***(-3.9, -1.2)^**^*** | | ***-1.8***  ***(-3.1, -0.6)^**^*** | | | ***-1.9***  ***(-2.6, -1.1)^***^*** | | ***-1.4***  ***(-2.2, -0.6)^**^*** | | ***0.9***  ***(-1.4, -0.6)^***^*** | | ***-0.9***  ***(-1.4, -0.5)^***^*** | |
| **UK Biobank** |  |  | | |  | |  | |  |  | |  | |  | |  | |  |
| **Bleeding gums (vs. healthy)** | | | ***0.9***  ***(0.8, 0.9) ^*^*** | ***0.9***  ***(0.7, 0.9) ^**^*** | | 0.9  (0.8, 1.2) | | 0.9  (0.8, 1.1) | | | 1.0  (0.9, 1.1) | | 0.9  (0.8, 1.1) | | **0.8**  **(0.7, 0.9) ^***^** | | **0.8**  **(0.7, 0.9) ^***^** | |
| **Painful gums (vs. healthy)** | | | ***0.8***  ***(0.7, 0.9) ^*^*** | ***0.8***  ***(0.6, 0.9) ^*^*** | | 1.1  (0.8, 1.5) | | 0.9  (0.7, 1.4) | | | 0.9  (0.7, 1.2) | | 0.9  (0.7, 1.2) | | **0.8**  **(0.6, 0.9) ^*^** | | **0.7**  **(0.6, 0.9) ^*^** | |
| **Loose teeth (vs. healthy)** | | | ***0.8***  ***(0.6, 0.9) ^*^*** | ***0.7***  ***(0.6, 0.9) ^**^*** | | ***0.7***  ***(0.5, 0.8) ^**^*** | | ***0.6***  ***(0.4, 0.8) ^***^*** | | | ***0.8***  ***(0.6, 0.9) ^*^*** | | ***0.7***  ***(0.5, 0.9) ^**^*** | | ***0.7***  ***(0.5, 0.9) ^**^*** | | ***0.6***  ***(0.5, 0.8) ^***^*** | |
| **Any periodontal disease (vs. healthy)** | | | ***0.7***  ***(0.5, 0.9) ^*^*** | ***0.7***  ***(0.5, 0.8) ^**^*** | | 0.9  (0.6, 1.0) | | ***0.7***  ***(0.5, 0.9) ^*^*** | | | 0.9  (0.7, 1.0) | | ***0.8***  ***(0.6, 0.9) ^*^*** | | ***0.7***  ***(0.5, 0.9) ^**^*** | | ***0.7***  ***(0.6, 0.9) ^**^*** | |

Abbreviations. CAL, clinical attachment level; CI, confidence interval; MD, difference in means; mm, millimeter; OR, odds ratio; PPD, probing pocket depth; %, percentage.

^*^ p<0.05; ^**^ p<0.01; ^***^ p<0.001.

**Table S3**. Association between categories of healthy lifestyle score (4-5 vs. 0-1 healthy lifestyles) and periodontitis by subgroups of age and sex.

| **4-5 healthy lifestyles (vs. 0-1 healthy lifestyles) as exposure – OR/MD (95% CI)** | | | | | | | | | | | | | | | | | | |
| --- | --- | --- | --- | --- | --- | --- | --- | --- | --- | --- | --- | --- | --- | --- | --- | --- | --- | --- |
| **Outcomes** | | | **Age** | | | | | | | | **Sex** | | | | | | | |
|  |  |  | **< 60 years** | | | **≥ 60 years** | | | | | **Males** | | | | **Females** | | | |
|  | | | ***Crude*** | ***Adjusted*** | | ***Crude*** | | ***Adjusted*** | | | ***Crude*** | | ***Adjusted*** | | ***Crude*** | | ***Adjusted*** | |
| **NHANES** |  |  | | |  | |  | |  |  | |  | |  | |  | |  |
| **Periodontitis** | | | ***0.2***  ***(0.1, 0.3)^***^*** | ***0.4***  ***(0.3, 0.5)^***^*** | | ***0.3***  ***(0.2, 0.5)^***^*** | | ***0.4***  ***(0.2, 0.6)^***^*** | | | ***0.3***  ***(0.2, 0.4)^***^*** | | ***0.4***  ***(0.3, 0.5)^***^*** | | ***0.4***  ***(0.3, 0.5)^***^*** | | ***0.4***  ***(0.3, 0.5)^***^*** | |
| **Periodontitis severity** | | |  |  | |  | |  | | |  | |  | |  | |  | |
| *Mild periodontitis* | | | ***0.4***  ***(0.3, 0.6)^***^*** | ***0.6***  ***(0.4, 0.9)^*^*** | | 1.7  (0.4, 7.4) | | 1.1  (0.2, 5.1) | | | ***0.4***  ***(0.2, 0.7)^**^*** | | 0.5  (0.3, 1.1) | | ***0.5***  ***(0.3, 0.9)^*^*** | | 0.7  (0.3, 1.1) | |
| *Moderate periodontitis* | | | ***0.3***  ***(0.2, 0.3)^***^*** | ***0.4***  ***(0.3, 0.5)^***^*** | | ***0.4***  ***(0.3, 0.7)^**^*** | | ***0.3***  ***(0.2, 0.9)^**^*** | | | ***0.4***  ***(0.3, 0.5)^***^*** | | ***0.3***  ***(0.2, 0.6)^**^*** | | ***0.5***  ***(0.4, 0.6)^***^*** | | ***0.5***  ***(0.3, 0.7)^**^*** | |
| *Severe periodontitis* | | | ***0.1***  ***(0.09, 0.2)^***^*** | ***0.2***  ***(0.1, 0.3)^***^*** | | ***0.1***  ***(0.08, 0.2)^***^*** | | ***0.1***  ***(0.1, 0.4)^***^*** | | | ***0.2***  ***(0.1, 0.2)^***^*** | | ***0.1***  ***(0.09, 0.4)^***^*** | | ***0.1***  ***(0.09, 0.2)^***^*** | | ***0.2***  ***(0.1, 0.3)^***^*** | |
| **PPD** | | |  |  | |  | |  | | |  | |  | |  | |  | |
| % sites PPD ≥ 4 mm | | | ***-3.2***  ***(-3.9, -2.6)^***^*** | ***-1.6***  ***(-2.2, -1.0)^***^*** | | ***-3.0***  ***(-4.5, -1.5)^***^*** | | ***-1.9***  ***(-3.3, -0.6)^**^*** | | | ***-3.7***  ***(-4.4, -2.9)^***^*** | | ***-1.7***  ***(-2.6, -0.7)^**^*** | | ***-2.3***  ***(-3.0, -1.6)^***^*** | | ***-1.6***  ***(-2.3, -0.8)^***^*** | |
| % sites PPD ≥ 5 mm | | | ***-1.1***  ***(-1.4, -0.8)^***^*** | ***-0.6***  ***(-0.8, -0.3)^***^*** | | ***-0.9***  ***(-1.6, -0.3)^**^*** | | -0.5  (-1.1, 0.1) | | | ***-1.2***  ***(-1.6, -0.8)^***^*** | | ***-0.5***  ***(-0.9, -0.04)^*^*** | | ***-0.7***  ***(-1.1, -0.4)^***^*** | | ***-0.5***  ***(-0.9, -0.2)^**^*** | |
| % sites PPD ≥ 6 mm | | | ***-0.4***  ***(-0.6, -0.2)^***^*** | ***-0.2***  ***(-0.3, -0.07)^**^*** | | -0.2  (-0.4, 0.009) | | 0.03  (-0.2, 0.3) | | | ***-0.4***  ***(-0.6, -0.2)^***^*** | | -0.1  (-0.3, 0.06) | | ***-0.3***  ***(-0.4, -0.1)^**^*** | | ***-0.2***  ***(-0.3, -0.04)^*^*** | |
| **CAL** | | |  |  | |  | |  | | |  | |  | |  | |  | |
| % sites CAL ≥ 3 mm | | | ***-10.3***  ***(-1..7, -8.8)^***^*** | ***-6.4***  ***(-7.9, -4.9)^***^*** | | ***-13.3***  ***(-16.9, -9.8)^***^*** | | ***-9.3***  ***(-12.2, -6.4)^***^*** | | | ***-11.1***  ***(-12.6, -9.6)^***^*** | | ***-7.9***  ***(-9.8, -5.9)^***^*** | | ***-7.1***  ***(-8.5, -5.7)^***^*** | | ***-5.7***  ***(-7.1, -4.4)^***^*** | |
| % sites CAL ≥ 4 mm | | | ***-6.4***  ***(-7.4, -5.3)^***^*** | ***-4.2***  ***(-5.3, -3.1)^***^*** | | ***-10.3***  ***(-12.9, -7.7)^***^*** | | ***-6.9***  ***(-9.1, -4.7)^***^*** | | | ***-7.6***  ***(-8.7, -6.4)^***^*** | | ***-5.4***  ***(-6.9, -3.9)^***^*** | | ***-4.6***  ***(-5.7, -3.5)^***^*** | | ***-3.8***  ***(-4.9, -2.7)^***^*** | |
| % sites CAL ≥ 5 mm | | | ***-3.6***  ***(-4.3, -2.9) ^***^*** | ***-2.4***  ***(-3.1, -1.7) ^***^*** | | ***-6.3***  ***(-8.1, -2.5) ^***^*** | | ***-3.9***  ***(-5.3, -2.4) ^***^*** | | | ***-4.7***  ***(-5.6, -3.8) ^***^*** | | ***-3.2***  ***(-4.3, -2.0) ^***^*** | | ***-2.4***  ***(-3.1, -1.8) ^***^*** | | ***-2.0***  ***(-2.8, -1.3) ^***^*** | |
| % sites CAL ≥ 6 mm | | | ***-2.2***  ***(-2.6, -1.7) ^***^*** | ***-1.4***  ***(-1.9, -0.9)^***^*** | | ***-3.7***  ***(-5.2, -2.2) ^***^*** | | ***-2.1***  ***(-3.3, -0.9) ^**^*** | | | ***-2.9***  ***(-3.7, -2.3) ^***^*** | | ***-1.9***  ***(-2.8, -1.1) ^***^*** | | ***-1.3***  ***(-1.7, -0.9) ^***^*** | | ***-1.0***  ***(-1.5, -0.6) ^***^*** | |
| **UK Biobank** |  |  | | |  | |  | |  |  | |  | |  | |  | |  |
| **Bleeding gums (vs. healthy)** | | | ***0.8***  ***(0.7, 0.9) ^***^*** | ***0.7***  ***(0.7, 0.8) ^***^*** | | 0.9  (0.8, 1.1) | | ***0.8***  ***(0.7, 0.9) ^*^*** | | | ***0.8***  ***(0.7, 0.9) ^**^*** | | ***0.8***  ***(0.7, 0.9) ^**^*** | | ***0.7***  ***(0.6, 0.8) ^***^*** | | ***0.7***  ***(0.6, 0.8) ^***^*** | |
| **Painful gums (vs. healthy)** | | | ***0.7***  ***(0.6, 0.9) ^**^*** | ***0.5***  ***(0.4, 0.7) ^***^*** | | 1.1  (0.8, 1.6) | | 0.9  (0.6, 1.3) | | | 0.9  (0.6, 1.2) | | ***0.7***  ***(0.5, 0.9) ^*^*** | | ***0.7***  ***(0.5, 0.9) ^**^*** | | ***0.6***  ***(0.4, 0.7) ^***^*** | |
| **Loose teeth (vs. healthy)** | | | ***0.7***  ***(0.6, 0.9) ^**^*** | ***0.5***  ***(0.4, 0.6) ^***^*** | | ***0.6***  ***(0.5, 0.8) ^**^*** | | ***0.6***  ***(0.4, 0.7) ^***^*** | | | ***0.8***  ***(0.6, 0.9) ^*^*** | | ***0.5***  ***(0.4, 0.7) ^***^*** | | ***0.7***  ***(0.5, 0.9) ^**^*** | | ***0.5***  ***(0.4, 0.7) ^***^*** | |
| **Any periodontal disease (vs. healthy)** | | | ***0.7***  ***(0.5, 0.9) ^**^*** | ***0.5***  ***(0.4, 0.7)^***^*** | | ***0.7***  ***(0.6, 0.9) ^*^*** | | ***0.7***  ***(0.3, 0.6) ^***^*** | | | ***0.8***  ***(0.6, 0.9) ^*^*** | | ***0.6***  ***(0.5, 0.8) ^***^*** | | ***0.7***  ***(0.5, 0.9) ^**^*** | | ***0.6***  ***(0.5, 0.8)^**^*** | |

Abbreviations. CAL, clinical attachment level; CI, confidence interval; MD, difference in means; mm, millimeter; OR, odds ratio; PPD, probing pocket depth; %, percentage.

^*^ p<0.05; ^**^ p<0.01; ^***^ p<0.001.

**Table S4**. Association between smoking and periodontitis overall and by subgroups of age and sex.

| **Non-smoking (vs. smoking) as exposure – OR/MD (95% CI)** | | | | | | | | | | | | | | | | | |
| --- | --- | --- | --- | --- | --- | --- | --- | --- | --- | --- | --- | --- | --- | --- | --- | --- | --- |
| **Outcomes** | **Overall** | | | **Age** | | | | | | | **Sex** | | | | | | |
|  |  |  |  | **< 60 years** | | | **≥ 60 years** | | | | **Males** | | | | **Females** | | |
|  | ***Crude*** | | ***Adjusted*** | ***Crude*** | ***Adjusted*** | | ***Crude*** | | ***Adjusted*** | | ***Crude*** | | ***Adjusted*** | | ***Crude*** | | ***Adjusted*** |
| **NHANES** | | | | | | | | | | | | | | | | | |
| **Periodontitis** | ***0.4***  ***(0.3,0.4)^***^*** | | ***0.4***  ***(0.3, 0.4) ^***^*** | ***0.3***  ***(0.2, 0.4)^***^*** | ***0.4***  ***(0.3, 0.5)^***^*** | | ***0.3***  ***(0.2, 0.4)^***^*** | | ***0.3***  ***(0.2, 0.4)^***^*** | | ***0.3***  ***(0.2, 0.4)^***^*** | | ***0.3***  ***(0.3, 0.4) ^***^*** | | ***0.4***  ***(0.3, 0.5)^***^*** | | ***0.4***  ***(0.3, 0.5)^***^*** |
| **Periodontitis severity** |  | |  |  |  | |  | |  | |  | |  | |  | |  |
| *Mild periodontitis* | ***0.6***  ***(0.5, 0.9)^**^*** | | ***0.8***  ***(0.6, 1.2)*** | ***0.6***  ***(0.4, 0.9)^**^*** | 0.8  (0.6, 1.2) | | 1.4  (0.4, 4.9) | | 1.3  (0.4, 3.1) | | 0.7  (0.5, 1.1) | | 0.9  (0.6, 1.6) | | ***0.6***  ***(0.3, 0.9)^*^*** | | 0.7  (0.4, 1.1) |
| *Moderate periodontitis* | ***0.4***  ***(0.4,0.5)^***^*** | | ***0.4***  ***(0.3, 0.4)^***^*** | ***0.3***  ***(0.3, 0.4)^***^*** | ***0.4***  ***(0.3, 0.5)^***^*** | | ***0.3***  ***(0.2, 0.5)^***^*** | | ***0.3***  ***(0.2, 0.5)^***^*** | | ***0.4***  ***(0.3, 0.5)^***^*** | | ***0.3***  ***(0.2, 0.4)^***^*** | | ***0.5***  ***(0.4, 0.6)^***^*** | | ***0.4***  ***(0.3, 0.5)^***^*** |
| *Severe periodontitis* | ***0.2***  ***(0.2,0.3)^***^*** | | ***0.2***  ***(0.1, 0.3)^***^*** | ***0.2***  ***(0.1, 0.2)^***^*** | ***0.2***  ***(0.1, 0.3) ^***^*** | | ***0.1***  ***(0.08, 0.2)^***^*** | | ***0.2***  ***(0.1, 0.3) ^***^*** | | ***0.2***  ***(0.1, 0.3)^***^*** | | ***0.2***  ***(0.1, 0.3) ^***^*** | | ***0.2***  ***(0.1, 0.3)^***^*** | | ***0.2***  ***(0.1, 0.3)^***^*** |
| **PPD** |  | |  |  |  | |  | |  | |  | |  | |  | |  |
| % sites PPD ≥ 4 mm | ***-3.1***  ***(-3.6,-2.5)^***^*** | | ***-2.2***  ***(-2.8, -1.5) ^***^*** | ***-2.9***  ***(-3.6, -2.4)^***^*** | ***-2.0***  ***(-2.7, -1.3) ^***^*** | | ***-3.7***  ***(-5.4, -1.9)^***^*** | | ***-2.9***  ***(-4.6, -1.2) ^**^*** | | ***-3.7***  ***(-4.4, -2.9)^***^*** | | ***-2.5***  ***(-3.4, -1.7)^***^*** | | ***-2.1***  ***(-2.8, -1.4)^***^*** | | ***-1.6***  ***(-2.4, -0.8)^***^*** |
| % sites PPD ≥ 5 mm | ***-0.9***  ***(-1.1,-0.6)^***^*** | | ***-0.6***  ***(-0.9, -0.3)^***^*** | ***-0.9***  ***(-1.2, -0.6)^***^*** | ***-0.6***  ***(-0.9, -0.3)^***^*** | | ***-0.9***  ***(-1.7, -0.2)^*^*** | | -0.7  (-1.4, 0.04) | | ***-1.1***  ***(-1.4, -0.8)^***^*** | | ***-0.7***  ***(-1.1, -0.3)^***^*** | | ***-0.6***  ***(-0.9, -0.3)^***^*** | | ***-0.5***  ***(-0.8, -0.2)^**^*** |
| % sites PPD ≥ 6 mm | ***-0.3***  ***(-0.4, -0.2)^***^*** | | ***-0.2***  ***(-0.3, -0.05)^**^*** | ***-0.3***  ***(-0.4, -0.2)^***^*** | ***-0.2***  ***(-0.3, -0.03)^*^*** | | -0.2  (-0.5, 0.002) | | -0.08  (-0.3, 0.2) | | ***-0.3***  ***(-0.5, -0.2)^***^*** | | ***-0.2***  ***(-0.4, -0.01)^*^*** | | ***-0.1***  ***(-0.3, 0.05)^**^*** | | -0.1  (-0.2, 0.002) |
| **CAL** |  | |  |  |  | |  | |  | |  | |  | |  | |  |
| % sites CAL ≥ 3 mm | ***-9.3***  ***(-10.2, -8.4)^***^*** | | ***-8.2***  ***(-9.3, -7.2)^***^*** | ***-9.7***  ***(-10.7, -8.7)^***^*** | ***-7.3***  ***(-8.5, -6.2)^***^*** | | ***-14.3***  ***(-17.8, -10.9)^***^*** | | ***-12.5***  ***(-15.6, -9.4)^***^*** | | ***-11.0***  ***(-12.3, -9.7)^***^*** | | ***-9.9***  ***(-11.4, -8.3)^***^*** | | ***-6.6***  ***(-7.9, -5.3)^***^*** | | ***-6.1***  ***(-7.6, -4.7)^***^*** |
| % sites CAL ≥ 4 mm | ***-6.3***  ***(-7.2, -5.5)^***^*** | | ***-5.7***  ***(-6.7, -4.8)^***^*** | ***-6.3***  ***(-7.1, -5.5)^***^*** | ***-5.0***  ***(-5.9, -4.2)^***^*** | | ***-10.9***  ***(-14.1, -7.7)^***^*** | | ***-9.3***  ***(-12.3, -6.4)^***^*** | | ***-7.9***  ***(-9.2, -6.7)^***^*** | | ***-7.3***  ***(-8.7, -5.8)^***^*** | | ***-1.9***  ***(-5.1, -2.9)^***^*** | | ***-3.8***  ***(-4.9, -2.6)^***^*** |
| % sites CAL ≥ 5 mm | ***-3.8***  ***(-4.4, -3.2)^***^*** | | ***-3.4***  ***(-4.1, -2.7)^***^*** | ***-3.7***  ***(-4.4, -3.1)^***^*** | ***-3.0***  ***(-3.7, -2.4)^***^*** | | ***-6.8***  ***(-9.3, -4.4)^***^*** | | ***-5.6***  ***(-7.9, -3.3)^***^*** | | ***-5.1***  ***(-6.1, -4.0)^***^*** | | ***-4.6***  ***(-5.9, -3.3)^***^*** | | ***-2.0***  ***(-2.7, -1.4)^***^*** | | ***-1.9***  ***(-2.7, -1.3)^***^*** |
| % sites CAL ≥ 6 mm | ***-2.3***  ***(-2.8, -1.9)^***^*** | | ***-2.1***  ***(-2.6, -1.6)^***^*** | ***-2.2***  ***(-2.6, -1.8)^***^*** | ***-1.8***  ***(-2.2, -1.3)^***^*** | | ***-4.5***  ***(-6.5, -2.5)^***^*** | | ***-3.7***  ***(-5.6, -1.8)^***^*** | | ***-3.3***  ***(-4.2, -2.5)^***^*** | | ***-2.9***  ***(-3.9, -1.9)^***^*** | | ***-1.0***  ***(-1.4, -0.7)^***^*** | | ***-0.9***  ***(-1.4, -0.6)^***^*** |
| **UK Biobank** | | | | | | | | | | | | | | | | | |
| **Bleeding gums (vs. healthy)** | | ***1.1***  ***(1.0, 1.2) ^**^*** | 1.0  (0.9, 1.1) | ***1.1***  ***(1.0, 1.1) ^*^*** | 1.0  (0.9, 1.1) | 1.1  (0.9, 1.2) | | 1.0  (0.9, 1.1) | | 1.1  (0.9, 1.2) | | 1.0  (0.9, 1.1) | | 1.0  (0.9, 1.1) | | 1.0  (0.9, 1.1) | |
| **Painful gums (vs. healthy)** | | ***0.8***  ***(0.7, 0.9) ^***^*** | ***0.7***  ***(0.6, 0.8) ^***^*** | ***0.7***  ***(0.6, 0.8) ^***^*** | ***0.7***  ***(0.6, 0.8) ^***^*** | 0.9  (0.8, 1.1) | | 0.9  (0.7, 1.1) | | ***0.7***  ***(0.6, 0.8) ^***^*** | | ***0.7***  ***(0.6, 0.8) ^***^*** | | ***0.8***  ***(0.7, 0.9) ^**^*** | | ***0.8***  ***(0.7, 0.9) ^***^*** | |
| **Loose teeth (vs. healthy)** | | ***0.4***  ***(0.3, 0.5) ^***^*** | ***0.4***  ***(0.3, 0.5) ^***^*** | ***0.4***  ***(0.3, 0.5) ^***^*** | ***0.3***  ***(0.2, 0.49^***^*** | ***0.6***  ***(0.4, 0.6) ^***^*** | | ***0.6***  ***(0.5, 0.6) ^***^*** | | ***0.4***  ***(0.3, 0.5) ^***^*** | | ***0.4***  ***(0.3, 0.5) ^***^*** | | ***0.5***  ***(0.4, 0.5) ^***^*** | | ***0.4***  ***(0.3, 0.5) ^***^*** | |
| **Any periodontal disease (vs. healthy)** | | 0.9  (0.7, 1.2) | ***0.6***  ***(0.4, 0.8) ^*^*** | 0.8  (0.5, 1.0) | ***0.7***  ***(0.6, 0.9) ^*^*** | 0.9  (0.7, 1.0) | | 0.7  (0.4, 1.2) | | ***0.5***  ***(0.3, 0.9) ^*^*** | | ***0.5***  ***(0.3, 0.8) ^**^*** | | 0.9  (0.6, 1.2) | | ***0.7***  ***(0.4, 0.9) ^**^*** | |

Abbreviations. CAL, clinical attachment level; CI, confidence interval; MD, difference in means; mm, millimeter; OR, odds ratio; PPD, probing pocket depth; %, percentage.

^*^ p<0.05; ^**^ p<0.01; ^***^ p<0.001.

**Table S5**. Association between alcohol drinking and periodontitis overall and by subgroups of age and sex.

| **Alcohol below the recommended limitations (vs. above the recommended limitations) as exposure – OR/MD (95% CI)** | | | | | | | | | | | | | | | | | | | | |
| --- | --- | --- | --- | --- | --- | --- | --- | --- | --- | --- | --- | --- | --- | --- | --- | --- | --- | --- | --- | --- |
| **Outcomes** | **Overall** | | | | **Age** | | | | | | | | **Sex** | | | | | | | |
|  |  |  |  |  | **< 60 years** | | | | **≥ 60 years** | | | | **Males** | | | | **Females** | | | |
|  | ***Crude*** | | ***Adjusted*** | | ***Crude*** | | ***Adjusted*** | | ***Crude*** | | ***Adjusted*** | | ***Crude*** | | ***Adjusted*** | | ***Crude*** | | ***Adjusted*** | |
| **NHANES** | | | | | | | | | | | | | | | | | | | | |
| **Periodontitis** | ***0.8***  ***(0.7, 0.9)^**^*** | | ***0.7***  ***(0.6, 0.8)^***^*** | | ***0.7***  ***(0.6, 0.8)^***^*** | | ***0.7***  ***(0.6, 0.8)^***^*** | | 0.8  (0.7, 1.1) | | 0.8  (0.7, 1.1) | | ***0.6***  ***(0.5, 0.7)^***^*** | | ***0.7***  ***(0.6, 0.8)^***^*** | | 1.1  (0.9, 1.3) | | 0.9  (0.7, 1.0) | |
| **Periodontitis severity** |  | |  | |  | |  | |  | |  | |  | |  | |  | |  | |
| *Mild periodontitis* | 0.9  (0.7, 1.1) | | 0.9  (0.8, 1.2) | | 0.9  (0.7, 1.1) | | 0.9  (0.7, 1.2) | | 1.3  (0.7, 2.5) | | 1.3  (0.7, 2.5) | | 0.8  (0.6, 1.1) | | 0.9  (0.6, 1.3) | | 1.0  (0.7, 1.5) | | 1.0  (0.7, 1.5) | |
| *Moderate periodontitis* | 0.9  (0.7, 1.0) | | ***0.7***  ***(0.6, 0.9)^***^*** | | ***0.7***  ***(0.6, 0.8)^***^*** | | ***0.7***  ***(0.6, 0.9)^**^*** | | 0.9  (0.7, 1.1) | | 0.9  (0.7, 1.1) | | ***0.7***  ***(0.6, 0.8)^***^*** | | ***0.6***  ***(0.5, 0.7) ^***^*** | | 1.2  (0.9, 1.4) | | 0.9  (0.7, 1.1) | |
| *Severe periodontitis* | ***0.6***  ***(0.5, 0.8)^***^*** | | ***0.6***  ***(0.4, 0.7)^***^*** | | ***0.6***  ***(0.4, 0.7)^***^*** | | ***0.6***  ***(0.4, 0.8)^***^*** | | ***0.6***  ***(0.4, 0.8)^***^*** | | ***0.7***  ***(0.4, 0.9)^*^*** | | ***0.5***  ***(0.4, 0.6) ^***^*** | | ***0.5***  ***(0.4, 0.6) ^***^*** | | 0.9  (0.7, 1.4) | | 0.8  (0.6, 1.2) | |
| **PPD** |  | |  | |  | |  | |  | |  | |  | |  | |  | |  | |
| % sites PPD ≥ 4 mm | ***-1.0***  ***(-1.4, -0.7) ^***^*** | | ***-0.7***  ***(-1.0, -0.3) ^***^*** | | ***-1.0***  ***(-1.4, 0.7) ^***^*** | | ***-0.7***  ***(-1.2, -0.3) ^**^*** | | ***-0.9***  ***(-1.5, -0.4) ^**^*** | | ***-0.6***  ***(-1.2, -0.04) ^*^*** | | ***-1.6***  ***(-2.1, -1.1) ^***^*** | | ***-0.9***  ***(-1.5, -0.3) ^**^*** | | ***-0.4***  ***(-0.7, -0.08) ^*^*** | | ***-0.3***  ***(-0.6, -0.04) ^*^*** | |
| % sites PPD ≥ 5 mm | ***-0.4***  ***(-0.5, -0.2) ^***^*** | | ***-0.3***  ***(-0.4, -0.1) ^**^*** | | ***-0.4***  ***(-0.6, -0.2) ^***^*** | | ***-0.3 (-0.5, -0.1) ^**^*** | | ***-0.3***  ***(-0.5, -0.07) ^**^*** | | -0.1  (-0.3, 0.08) | | ***-0.6***  ***(-0.8, -0.3) ^***^*** | | ***-0.3***  ***(-0.6, -0.07) ^*^*** | | -0.1  (-0.3, 0.03) | | -0.1  (-0.3, 0.03) | |
| % sites PPD ≥ 6 mm | -0.1  (-0.2, -0.05) | | ***-0.1***  ***(-0.2, -0.03) ^**^*** | | ***-0.2***  ***(-0.1, -0.06) ^**^*** | | ***-0.1***  ***(-0.2, -0.04) ^**^*** | | -0.06  (-0.2, 0.03) | | 0.03  (-0.06, 0.1) | | ***-0.2***  ***(-0.3, -0.08) ^**^*** | | ***-0.2***  ***(-0.3, -0.02) ^*^*** | | -0.04  (-0.1, 0.03) | | -0.05  (-0.1, 0.03) | |
| **CAL** |  | |  | |  | |  | |  | |  | |  | |  | |  | |  | |
| % sites CAL ≥ 3 mm | ***-2.2***  ***(-3.1, 1.4) ^***^*** | | ***-2.6***  ***(-3.4, -1.8) ^***^*** | | ***-3.0***  ***(-3.9, -2.1) ^***^*** | | ***-2.5***  ***(-3.4, -1.5) ^***^*** | | ***-3.7***  ***(-6.2, -1.2) ^**^*** | | ***-2.5***  ***(-4.7, -0.3) ^*^*** | | ***-4.2***  ***(-5.5, -2.9) ^***^*** | | ***-3.8***  ***(-5.0, -2.5) ^***^*** | | 0.03  (-0.9, 0.9) | | ***-1.1***  ***(-1.8, -0.3) ^**^*** | |
| % sites CAL ≥ 4 mm | ***-1.6***  ***(-2.3, -1.0) ^***^*** | | ***-1.9***  ***(-2.4, -1.3) ^***^*** | | ***-2.0***  ***(-2.7, -1.4) ^***^*** | | ***-1.7***  ***(-2.4, -1.1) ^***^*** | | ***-2.9***  ***(-4.6, 1.1) ^**^*** | | ***-1.9***  ***(-3.5, -0.2) ^*^*** | | ***-2.8***  ***(-3.8, -1.9) ^***^*** | | ***-2.5***  ***(-3.6, -1.5) ^***^*** | | -0.3  (-0.9, 0.4) | | ***-0.9***  ***(-1.4, -0.3) ^**^*** | |
| % sites CAL ≥ 5 mm | ***-1.0***  ***(-1.5, -0.6) ^***^*** | | ***-1.1***  ***(-1.6, -0.7) ^***^*** | | ***-1.2***  ***(-1.7, -0.8) ^***^*** | | ***-1.1***  ***(-1.5, -0.6) ^***^*** | | ***-1.9***  ***(-3.0, -0.7) ^**^*** | | -1.1  (-2.2, 0.04) | | ***-1.8***  ***(-2.5, -1.1) ^***^*** | | ***-1.6***  ***(-2.5, -0.8) ^***^*** | | -0.2  (-0.5, 0.2) | | ***-0.4***  ***(-0.8, -0.1) ^*^*** | |
| % sites CAL ≥ 6 mm | ***-0.7***  ***(-1.0, -0.4) ^***^*** | | ***-0.8***  ***(-1.1, -0.4) ^***^*** | | ***-0.8***  ***(-1.1, -0.5) ^***^*** | | ***-0.7***  ***(-0.9, -0.4) ^***^*** | | ***-1.3***  ***(-2.1, -0.4) ^**^*** | | -0.8  (-1.6, 0.1) | | -1.3  (-1.8, -0.7) ^***^ | | -1.1  (-1.8, -0.5) | | -0.08  (-0.3, 0.1) | | ***-0.2***  ***(-0.4, -0.01) ^*^*** | |
| **UK Biobank** | | | | | | | | | | | | | | | | | | | | |
| **Bleeding gums (vs. healthy)** | | 0.9  (0.9, 1.0) | | ***0.8***  ***(0.8, 0.9) ^***^*** | | 1.0  (0.9, 1.1) | | ***0.9***  ***(0.8, 0.9) ^***^*** | | 1.0  (0.9, 1.1) | | ***0.9***  ***(0.8, 0.9) ^**^*** | | ***0.9***  ***(0.8, 0.9) ^*^*** | | ***0.9***  ***(0.8, 0.9) ^**^*** | | ***0.9***  ***(0.8, 0.9) ^***^*** | | ***0.9***  ***(0.8, 0.9) ^***^*** |
| **Painful gums (vs. healthy)** | | ***0.7***  ***(0.6, 0.8) ^***^*** | | 1.0  (0.9, 1.2) | | ***0.6***  ***(0.5, 0.8) ^**^*** | | 1.0  (0.9, 1.2) | | ***0.7***  ***(0.6, 0.8) ^**^*** | | 1.1  (0.9, 1.4) | | ***0.7***  ***(0.6, 0.8) ^**^*** | | 1.1  (0.9, 1.3) | | 1.2  (0.9, 1.3) | | 1.0  (0.9, 1.1) |
| **Loose teeth (vs. healthy)** | | ***0.6***  ***(0.5, 0.7) ^***^*** | | 0.9  (0.8, 1.1) | | ***0.5***  ***(0.5, 0.7) ^***^*** | | 1.1  (0.9, 1.3) | | 1.1  (0.9, 1.3) | | 0.9  (0.8, 1.1) | | ***0.6***  ***(0.5, 0.8) ^**^*** | | 1.1  (0.9, 1.2) | | ***0.7***  ***(0.6, 0.8) ^**^*** | | 0.9  (0.8, 1.1) |
| **Any periodontal disease (vs. healthy)** | | ***0.8***  ***(0.5, 0.9) ^*^*** | | 1.1  (0.9, 1.3) | | ***0.7***  ***(0.4, 0.9) ^*^*** | | 1.0  (0.9, 1.2) | | ***0.8***  ***(0.6, 0.9) ^*^*** | | 1.0  (0.9, 1.3) | | ***0.8***  ***(0.6, 0.9) ^*^*** | | 1.0  (0.8, 1.2) | | 1.0  (0.8, 1.2) | | 0.8  (0.7, 1.2) |

Abbreviations. CAL, clinical attachment level; CI, confidence interval; MD, difference in means; mm, millimeter; OR, odds ratio; PPD, probing pocket depth; %, percentage.

^*^ p<0.05; ^**^ p<0.01; ^***^ p<0.001.

**Table S6**. Association between leisure-time physical activity and periodontitis overall and by subgroups of age and sex.

| **High LTPA (vs. low LTPA) as exposure – OR/MD (95% CI)** | | | | | | | | | | | | | | | | | | | | | | | |
| --- | --- | --- | --- | --- | --- | --- | --- | --- | --- | --- | --- | --- | --- | --- | --- | --- | --- | --- | --- | --- | --- | --- | --- |
| **Outcomes** | | **Overall** | | | | **Age** | | | | | | | | **Sex** | | | | | | | | | |
|  |  |  |  |  |  | **< 60 years** | | | | **≥ 60 years** | | | | **Males** | | | | **Females** | | | | | |
|  | | ***Crude*** | | ***Adjusted*** | | ***Crude*** | | ***Adjusted*** | | ***Crude*** | | ***Adjusted*** | | ***Crude*** | | ***Adjusted*** | | ***Crude*** | | ***Adjusted*** | | |  |
| **NHANES** | | | | | | | | | | | | | | | | | | | | | | |  |
| **Periodontitis** | | ***0.6***  ***(0.5, 0.6)^***^*** | | ***0.7***  ***(0.6, 0.8)^***^*** | | ***0.6***  ***(0.5, 0.7)^***^*** | | ***0.7***  ***(0.6, 0.8)^***^*** | | ***0.7***  ***(0.5, 0.8)^**^*** | | ***0.7***  ***(0.6, 0.9)^*^*** | | ***0.5***  ***(0.4, 0.6)^***^*** | | ***0.7***  ***(0.6, 0.8)^***^*** | | ***0.6***  ***(0.5, 0.7)^***^*** | | ***0.8***  ***(0.7, 0.9)^**^*** | | |  |
| **Periodontitis severity** | |  | |  | |  | |  | |  | |  | |  | |  | |  | |  | | |  |
| *Mild periodontitis* | | ***0.6***  ***(0.5, 0.8)^**^*** | | 0.8  (0.6, 1.0) | | ***0.6***  ***(0.5, 0.8)^**^*** | | 0.8  (0.6, 1.1) | | 0.7  (0.4, 1.2) | | 0.7  (0.4, 1.3) | | ***0.5***  ***(0.3, 0.7)^***^*** | | ***0.6***  ***(0.4, 0.9)^*^*** | | 0.7  (0.5, 1.1) | | 1.0  (0.7, 1.5) | | |  |
| *Moderate periodontitis* | | ***0.6***  ***(0.5, 0.7)^***^*** | | ***0.8***  ***(0.7, 0.9)^**^*** | | ***0.6***  ***(0.5, 0.7)^***^*** | | ***0.8***  ***(0.7, 0.9)^*^*** | | ***0.7***  ***(0.5, 0.9)^**^*** | | 0.8  (0.6, 1.0) | | ***0.6***  ***(0.5, 0.7)^***^*** | | ***0.7***  ***(0.6, 0.9)^**^*** | | ***0.6***  ***(0.5, 0.7)^***^*** | | ***0.8***  ***(0.7, 0.9)^**^*** | | |  |
| *Severe periodontitis* | | ***0.4***  ***(0.3, 0.5)^***^*** | | ***0.6***  ***(0.5, 0.8)^***^*** | | ***0.4***  ***(0.3, 0.5)^***^*** | | ***0.6***  ***(0.4, 0.8)^**^*** | | ***0.6***  ***(0.4, 0.8)^**^*** | | 0.8  (0.6, 1.1) | | ***0.4***  ***(0.3, 0.5)^***^*** | | ***0.5***  ***(0.4, 0.7)^***^*** | | ***0.4***  ***(0.3, 0.6)^***^*** | | 0.6  (0.4, 0.9) | | |  |
| **PPD** | |  | |  | |  | |  | |  | |  | |  | |  | |  | |  | | |  |
| % sites PPD ≥ 4 mm | | ***-1.2***  ***(-1.5, -0.9)^***^*** | | ***-0.8***  ***(-1.0, -0.5)^***^*** | | ***-1.4***  ***(-1.8, 1.1)^***^*** | | ***-0.9***  ***(-1.3, -0.6)^***^*** | | ***-0.5***  ***(-0.9, -0.03)^*^*** | | -0.1  (-0.6, 0.4) | | ***-1.8***  ***(-2.2, -1.3)^***^*** | | ***-1.1***  ***(-1.6, -0.7) ^***^*** | | ***-0.9***  ***(-1.2, -0.6)^***^*** | | ***-0.4***  ***(-0.7, -0.09)^*^*** | | |  |
| % sites PPD ≥ 5 mm | | ***-0.4***  ***(-0.5, -0.2)^***^*** | | ***-0.2***  ***(-0.4, -0.1)^***^*** | | ***-0.5***  ***(-0.6, -0.3)^***^*** | | ***-0.3***  ***(-0.5, -0.2)^***^*** | | -0.1  (-0.4, 0.08) | | 0.003  (-0.2, 0.3) | | ***-0.6***  ***(-0.8, -0.4)^***^*** | | ***-0.4***  ***(-0.6, -0.2)^***^*** | | ***-0.2***  ***(-0.4, -0.08)^**^*** | | -0.09  (-0.2, 0.06) | | |  |
| % sites PPD ≥ 6 mm | | ***-0.1***  ***(-0.2, -0.04)^**^*** | | -0.06  (-0.1, 0.005) | | -0.2  (-0.2, -0.07)^***^ | | -0.09  (-0.2, -0.02)^*^ | | 0.02  (-0.1, 0.1) | | 0.08  (-0.06, 0.2) | | ***-0.2***  ***(-0.3, -0.1)^***^*** | | ***-0.1***  ***(-0.2, -0.02)^*^*** | | -0.06  (-0.1, 0.01) | | 0.001  (-0.07, 0.07) | | |  |
| **CAL** | |  | |  | |  | |  | |  | |  | |  | |  | |  | |  | | |  |
| % sites CAL ≥ 3 mm | | ***-4.2***  ***(-4.8, -3.6)^***^*** | | ***-2.0***  ***(-2.6, -1.4)^***^*** | | ***-3.8***  ***(-4.6, -3.1)^***^*** | | ***-1.9***  ***(-2.8, -1.2)^***^*** | | ***-4.1***  ***(-5.6, -2.6)^***^*** | | ***-2.0***  ***(-3.4, -0.6)^**^*** | | ***-5.6***  ***(-6.7, -4.7)^***^*** | | ***-2.8***  ***(-3.8, -1.8)^***^*** | | ***-3.6***  ***(-4.5, -2.7)^***^*** | | ***-1.3***  ***(-2.1, -0.5)^**^*** | | |  |
| % sites CAL ≥ 4 mm | | ***-2.7***  ***(-3.2, -2.2)^***^*** | | ***-1.3***  ***(-1.7, -0.9)^***^*** | | ***-2.3***  ***(-2.8, -1.8)^***^*** | | ***-1.2***  ***(-1.7, -0.8)^***^*** | | ***-3.1***  ***(-4.3, -1.9)^***^*** | | ***-1.4***  ***(-2.5, -0.3)^*^*** | | ***-3.7***  ***(-4.5, -2.9)^***^*** | | ***-1.8***  ***(-2.5, -1.1)^***^*** | | ***-2.1***  ***(-2.7, -1.6)^***^*** | | ***-0.8***  ***(-1.3, -0.2)^**^*** | | |  |
| % sites CAL ≥ 5 mm | | ***-1.5***  ***(-1.8, -1.2)^***^*** | | ***-0.7***  ***(-0.9, -0.4)^***^*** | | ***-1.2***  ***(-1.6, -0.9)^***^*** | | ***-0.7***  ***(-1.0, -0.3)^***^*** | | ***-1.8***  ***(-2.6, -0.9)^***^*** | | -0.6  (-1.4, 0.09) | | ***-2.2***  ***(-2.7, -1.6)^***^*** | | ***-0.9***  ***(-1.5, -0.5)^***^*** | | ***-1.1***  ***(-1.4, -0.7)^***^*** | | ***-0.4***  ***(-0.7, -0.03)^*^*** | | |  |
| % sites CAL ≥ 6 mm | | ***-0.8***  ***(-1.1, -0.6)^***^*** | | ***-0.4***  ***(-0.6, -0.2)^***^*** | | ***-0.7***  ***(-0.9, -0.5)^***^*** | | ***-0.4***  ***(-0.6, -0.2)^**^*** | | ***-0.9***  ***(-1.6, -0.4)^**^*** | | -0.2  (-0.8, 0.3) | | ***-1.3***  ***(-1.6, -0.9)^***^*** | | ***-0.5***  ***(-0.9, -0.2)^**^*** | | ***-0.6***  ***(-0.8, -0.4)^***^*** | | ***-0.2***  ***(-0.4, -0.01)^*^*** | | |  |
| **UK Biobank** | | | | | | | | | | | | | | | | | | | | | |  |  |
| **Bleeding gums (vs. healthy)** | ***0.9***  ***(0.8, 0.9) ^***^*** | | ***0.9***  ***(0.8, 0.9) ^***^*** | | ***0.9***  ***(0.8, 0.9) ^**^*** | | ***0.9***  ***(0.8, 0.9) ^***^*** | | 0.9  (0.8, 1.0) | | 0.9  (0.8, 1.0) | | 0.9  (0.8, 1.0) | | 0.9  (0.8, 1.0) | | 1.0  (0.9, 1.2) | | ***0.9***  ***(0.8, 0.9) ^***^*** | |  |  |  |
| **Painful gums (vs. healthy)** | ***1.1***  ***(1.0, 1.2) ^*^*** | | 0.9  (0.8, 1.1) | | 1.1  (0.9, 1.2) | | 0.9  (0.8, 1.1) | | 1.2  (0.9, 1.4) | | 1.0  (0.8, 1.2) | | ***1.2***  ***(1.0, 1.5) ^*^*** | | 1.1  (0.9, 1.3) | | 1.1  (0.9, 1.2) | | 0.9  (0.8, 1.1) | |  |  |  |
| **Loose teeth (vs. healthy)** | ***0.7***  ***(0.7, 0.8) ^***^*** | | 1.1  (0.9, 1.2) | | ***0.7***  ***(0.6, 0.8) ^***^*** | | ***0.8***  ***(0.7, 0.9) ^**^*** | | ***0.8***  ***(0.7, 0.9) ^*^*** | | 1.0  (0.9, 1.2) | | ***0.8***  ***(0.8, 0.9) ^*^*** | | 1.0  (0.9, 1.3) | | ***0.7***  ***(0.6, 0.8) ^***^*** | | 1.1  (0.9, 1.3) | |  |  |  |
| **Any periodontal disease (vs. healthy)** | ***0.8***  ***(0.7, 0.9) ^*^*** | | ***0.8***  ***(0.6, 0.9) ^**^*** | | ***0.7***  ***(0.6, 0.9) ^**^*** | | 0.9  (0.7, 1.0) | | 0.9  (0.8, 1.1) | | 0.9  (0.8, 1.2) | | ***0.8***  ***(0.8, 0.9) ^*^*** | | 0.9  (0.8, 1.1) | | 0.9  (0.8, 1.0) | | ***0.9***  ***(0.7, 0.9) ^**^*** | |  |  |  |

Abbreviations. CAL, clinical attachment level; CI, confidence interval; LTPA, leisure-time physical activity; MD, difference in means; mm, millimeter; OR, odds ratio; PPD, probing pocket depth; %, percentage.

^*^ p<0.05; ^**^ p<0.01; ^***^ p<0.001.

**Table S7**. Association between diet quality and periodontitis overall and by subgroups of age and sex.

| **Healthy diet (vs. unhealthy diet) as exposure – OR/MD (95% CI)** | | | | | | | | | | | | | | | | | | | | | | | |
| --- | --- | --- | --- | --- | --- | --- | --- | --- | --- | --- | --- | --- | --- | --- | --- | --- | --- | --- | --- | --- | --- | --- | --- |
| **Outcomes** | | **Overall** | | | | **Age** | | | | | | | | **Sex** | | | | | | | | | |
|  |  |  |  |  |  | **< 60 years** | | | | **≥ 60 years** | | | | **Males** | | | | **Females** | | | | |  |
|  | | ***Crude*** | | ***Adjusted*** | | ***Crude*** | | ***Adjusted*** | | ***Crude*** | | ***Adjusted*** | | ***Crude*** | | ***Adjusted*** | | ***Crude*** | | ***Adjusted*** | | |  |
| **NHANES** | | | | | | | | | | | | | | | | | | | | | | |  |
| **Periodontitis** | | ***0.8***  ***(0.7, 0.9)^***^*** | | ***0.8***  ***(0.7, 0.9)^***^*** | | ***0.7***  ***(0.6, 0.8)^***^*** | | ***0.8***  ***(0.7, 0.9)^**^*** | | ***0.8***  ***(0.7, 0.9)^**^*** | | 0.8  (0.7, 1.0) | | 0.9  (0.8, 1.0) | | 0.9  (0.7, 1.0) | | ***0.8***  ***(0.7, 0.9)^**^*** | | ***0.7***  ***(0.6, 0.9)^***^*** | | |  |
| **Periodontitis severity** | |  | |  | |  | |  | |  | |  | |  | |  | |  | |  | | |  |
| *Mild periodontitis* | | ***0.7***  ***(0.6, 0.8)^**^*** | | ***0.8***  ***(0.6, 0.9)^*^*** | | ***0.7***  ***(0.6, 0.9)^*^*** | | 0.9  (0.7, 1.1) | | 0.6  (0.4, 1.1) | | ***0.5***  ***(0.3, 0.9) ^*^*** | | ***0.6***  ***(0.4, 0.9)^*^*** | | 0.7  (0.5, 1.2) | | 0.8  (0.6, 1.2) | | 0.9  (0.6, 1.3) | | |  |
| *Moderate periodontitis* | | ***0.9***  ***(0.8, 0.9)^**^*** | | ***0.8***  ***(0.7, 0.9)^**^*** | | ***0.7***  ***(0.6, 0.8)^***^*** | | ***0.8***  ***(0.7, 0.9)^**^*** | | 0.9  (0.7, 1.0) | | 0.9  (0.7, 1.1) | | 0.9  (0.8, 1.1) | | 0.9  (0.8, 1.1) | | ***0.8***  ***(0.7, 0.9)^**^*** | | ***0.8***  ***(0.7, 0.9)^**^*** | | |  |
| *Severe periodontitis* | | ***0.6***  ***(0.5, 0.8)^***^*** | | ***0.7***  ***(0.6, 0.9)^**^*** | | ***0.6***  ***(0.5, 0.8)^***^*** | | ***0.7***  ***(0.6, 0.9)^*^*** | | ***0.6***  ***(0.4, 0.8)^***^*** | | ***0.7***  ***(0.5, 0.9)^*^*** | | 0.8  (0.7, 1.0) | | 0.8  (0.6, 1.0) | | ***0.5***  ***(0.3, 0.6)^***^*** | | ***0.5***  ***(0.3, 0.7)^***^*** | | |  |
| **PPD** | |  | |  | |  | |  | |  | |  | |  | |  | |  | |  | | |  |
| % sites PPD ≥ 4 mm | | ***-0.8***  ***(-1.1, -0.5)^***^*** | | ***-0.3***  ***(-0.7, -0.01)^*^*** | | ***-0.9***  ***(-1.2, -0.6)^***^*** | | ***-0.4***  ***(-0.8, -0.01)^*^*** | | ***-0.6***  ***(-1.0, -0.1)^*^*** | | -0.2  (-0.6, 0.2) | | ***-0.7***  ***(-1.2, -0.2)^**^*** | | -0.2  (-0.7, 0.4) | | ***-0.7***  ***(-0.9, -0.4)^***^*** | | ***-0.5***  ***(-0.8, -0.2)^**^*** | | |  |
| % sites PPD ≥ 5 mm | | ***-0.3***  ***(-0.4, -0.1)^***^*** | | -0.1  (-0.2, 0.03) | | ***-0.31***  ***(-0.5, -0.1)^**^*** | | -0.1  (-0.3, 0.03) | | -0.2  (-0.4, 0.001) | | -0.05  (-0.2, 0.1) | | -0.2  (-0.5, 0.01) | | -0.04  (-0.3, 0.2) | | ***-0.3***  ***(-0.4, -0.1)^***^*** | | ***-0.2***  ***(-0.3, -0.06)^**^*** | | |  |
| % sites PPD ≥ 6 mm | | ***-0.09***  ***(-0.2, -0.04)^**^*** | | -0.04  (-0.1, 0.02) | | ***-0.1***  ***(-0.2, -0.03)^**^*** | | -0.06  (-0.1, 0.02) | | -0.05  (-0.2, 0.06) | | 0.03  (-0.08, 0.1) | | -0.06  (-0.2, 0.05) | | -0.01  (-0.1, 0.1) | | ***-0.1***  ***(-0.2, -0.04)^**^*** | | ***-0.08***  ***(-0.1, -0.02)^**^*** | | |  |
| **CAL** | |  | |  | |  | |  | |  | |  | |  | |  | |  | |  | | |  |
| % sites CAL ≥ 3 mm | | ***-1.6***  ***(-2.4, -0.9)^***^*** | | ***-1.3***  ***(-2.0, -0.5)^**^*** | | ***-2.2***  ***(-2.9, -1.4)^***^*** | | ***-1.2***  ***(-1.9, -0.5)^**^*** | | ***-2.5***  ***(-4.3, -0.8)^**^*** | | -1.3  (-2.8, 0.2) | | -0.8  (-1.9, 0.3) | | -0.9  (-2.1, 0.1) | | ***-1.8***  ***(-2.6, -0.9)^***^*** | | ***-1.7***  ***(-2.4, -0.9)^***^*** | | |  |
| % sites CAL ≥ 4 mm | | ***-1.0***  ***(-1.5, -0.5)^***^*** | | ***-0.8***  ***(-1.3, -0.3)^**^*** | | ***-1.1***  ***(-1.6, -0.6)^***^*** | | ***-0.6***  ***(-1.1, -0.1)^*^*** | | ***-2.1***  ***(-3.5, -0.7)^**^*** | | -1.0  (-2.1, 0.09) | | -0.6  (1.4, 0.2) | | -0.7  (-1.5, 0.09) | | ***-0.9***  ***(-1.4, -0.5)^***^*** | | ***-0.9***  ***(-1.4, -0.4)^***^*** | | |  |
| % sites CAL ≥ 5 mm | | ***-0.6***  ***(-0.9, -0.3)^**^*** | | ***-0.5***  ***(-0.8, -0.1)^**^*** | | ***-0.6***  ***(-1.0, -0.2)^**^*** | | -0.4  (-0.7, 0.03) | | ***-1.4***  ***(-2.4, -0.4)^**^*** | | -0.6  (-1.4, 0.3) | | -0.5  (1.1, 0.2) | | -0.5  (-1.1, 0.2) | | ***-0.5***  ***(-0.8, -0.3)^***^*** | | ***-0.5***  ***(-0.8, -0.2)^**^*** | | |  |
| % sites CAL ≥ 6 mm | | ***-0.4***  ***(-0.7, -0.1)^**^*** | | ***-0.3***  ***(-0.5, -0.02)^*^*** | | ***-0.4***  ***(-0.7, -0.1)^**^*** | | -0.2  (-0.5, 0.03) | | ***-0.8***  ***(-1.5, -0.02)^*^*** | | -0.2  (-0.9, 0.4) | | -0.3  (-0.8, 0.1) | | -0.3  (-0.7, 0.2) | | ***-0.3***  ***(-0.5, -0.1)^**^*** | | ***-0.3***  ***(-0.5, -0.1)^**^*** | | |  |
| **UK Biobank** | | | | | | | | | | | | | | | | | | | | | |  |  |
| **Bleeding gums (vs. healthy)** | ***0.9***  ***(0.8, 0.9) ^***^*** | | ***0.8***  ***(0.7, 0.9) ^***^*** | | ***0.8***  ***(0.7, 0.9) ^***^*** | | ***0.8***  ***(0.7, 0.9) ^***^*** | | 0.9  (0.8, 1.0) | | 0.9  (0.8, 1.0) | | ***0.8***  ***(0.7, 0.9) ^***^*** | | ***0.8***  ***(0.7, 0.9) ^***^*** | | ***0.9***  ***(0.8, 0.9) ^***^*** | | ***0.9***  ***(0.8, 0.9) ^***^*** | |  |  |  |
| **Painful gums (vs. healthy)** | ***1.1***  ***(1.0, 1.3) ^*^*** | | 0.9  (0.9, 1.1) | | 1.1  (0.9, 1.3) | | 0.9  (0.8, 1.0) | | ***1.2***  ***(1.0, 1.5) ^*^*** | | 1.1  (0.9, 1.4) | | 1.1  (0.9, 1.3) | | 1.0  (0.8, 1.3) | | 1.1  (0.9, 1.3) | | 0.9  (0.8, 1.2) | |  |  |  |
| **Loose teeth (vs. healthy)** | ***0.8***  ***(0.7, 0.9) ^**^*** | | 1.0  (0.9, 1.2) | | ***0.8***  ***(0.8, 0.9) ^*^*** | | 1.0  (0.8, 1.2) | | 1.1  (0.9, 1.3) | | 1.0  (0.9, 1.3) | | ***0.8***  ***(0.7, 0.8) ^*^*** | | 1.1  (0.9, 1.3) | | 1.1  (0.9, 1.3) | | 1.0  (0.9, 1.2) | |  |  |  |
| **Any periodontal disease (vs. healthy)** | ***0.9***  ***(0.8, 0.9)^**^*** | | ***0.8***  ***(0.7, 0.9) ^***^*** | | ***0.8***  ***(0.7, 0.9) ^***^*** | | ***0.8***  ***(0.7, 0.9) ^***^*** | | 0.9  (0.8, 1.0) | | 0.9  (0.8, 1.0) | | ***0.8***  ***(0.7, 0.9) ^***^*** | | ***0.8***  ***(0.7, 0.9) ^***^*** | | ***0.9***  ***(0.8, 0.9) ^***^*** | | ***0.9***  ***(0.8, 0.9) ^**^*** | |  |  |  |

Abbreviations. CAL, clinical attachment level; CI, confidence interval; MD, difference in means; mm, millimeter; OR, odds ratio; PPD, probing pocket depth; %, percentage.

^*^ p<0.05; ^**^ p<0.01; ^***^ p<0.001.

**Table S8**. Association between sleep duration and periodontitis overall and by subgroups of age and sex.

| **Proper sleep duration (vs. short/long sleep duration) as exposure – OR/MD (95% CI)** | | | | | | | | | | | | | | | | | | | | | | | |
| --- | --- | --- | --- | --- | --- | --- | --- | --- | --- | --- | --- | --- | --- | --- | --- | --- | --- | --- | --- | --- | --- | --- | --- |
| **Outcomes** | | **Overall** | | | | **Age** | | | | | | | | **Sex** | | | | | | | | | |
|  |  |  |  |  |  | **< 60 years** | | | | **≥ 60 years** | | | | **Males** | | | | **Females** | | | | | |
|  | | ***Crude*** | | ***Adjusted*** | | ***Crude*** | | ***Adjusted*** | | ***Crude*** | | ***Adjusted*** | | ***Crude*** | | ***Adjusted*** | | ***Crude*** | | ***Adjusted*** | | |  |
| **NHANES** | | | | | | | | | | | | | | | | | | | | | | |  |
| **Periodontitis** | | ***0.7***  ***(0.6, 0.8)^***^*** | | ***0.8***  ***(0.7, 0.8)^***^*** | | ***0.7***  ***(0.6, 0.7)^***^*** | | ***0.7***  ***(0.6, 0.8)^***^*** | | ***0.8***  ***(0.6, 0.9)^**^*** | | ***0.8***  ***(0.7, 0.9)^*^*** | | ***0.8***  ***(0.7, 0.9)^***^*** | | ***0.7***  ***(0.6, 0.9)^**^*** | | ***0.7***  ***(0.6, 0.8)^***^*** | | ***0.8***  ***(0.7, 0.9)^**^*** | | |  |
| **Periodontitis severity** | |  | |  | |  | |  | |  | |  | |  | |  | |  | |  | | |  |
| *Mild periodontitis* | | ***0.7***  ***(0.6, 0.9)^*^*** | | 0.8  (0.6, 1.0) | | ***0.7***  ***(0.6, 0.9)^*^*** | | 0.8  (0.6, 1.0) | | 0.8  (0.4, 1.7) | | 0.8  (0.4, 1.6) | | 0.8  (0.6, 1.2) | | 0.9  (0.6, 1.2) | | ***0.6***  ***(0.4, 0.9)^*^*** | | ***0.7***  ***(0.5, 0.9)^*^*** | | |  |
| *Moderate periodontitis* | | ***0.7***  ***(0.7, 0.8)^***^*** | | ***0.8***  ***(0.7, 0.9)^***^*** | | ***0.7***  ***(0.6, 0.8)^***^*** | | ***0.7***  ***(0.6, 0.9)^***^*** | | ***0.8***  ***(0.6, 0.9)^*^*** | | 0.8  (0.7, 1.1) | | ***0.8***  ***(0.6, 0.9)^**^*** | | ***0.7***  ***(0.6, 0.9)^**^*** | | ***0.7***  ***(0.6, 0.9)^***^*** | | 0.9  (0.7, 1.0) | | |  |
| *Severe periodontitis* | | ***0.6***  ***(0.5, 0.7)^***^*** | | ***0.7***  ***(0.6, 0.8)^***^*** | | ***0.6***  ***(0.5, 0.7)^***^*** | | ***0.7***  ***(0.6, 0.9)^**^*** | | ***0.6***  ***(0.5, 0.8)^**^*** | | ***0.7***  ***(0.5, 0.9)^**^*** | | ***0.8***  ***(0.6, 0.9)^*^*** | | ***0.7***  ***(0.6, 0.9)^*^*** | | ***0.4***  ***(0.3, 0.5)^***^*** | | ***0.5***  ***(0.4, 0.6)^***^*** | | |  |
| **PPD** | |  | |  | |  | |  | |  | |  | |  | |  | |  | |  | | |  |
| % sites PPD ≥ 4 mm | | ***-0.5***  ***(-0.8, -0.3)^***^*** | | -0.2  (-0.4, 0.09) | | ***-0.5***  ***(-0.8, -0.2)^**^*** | | -0.09  (-0.4, 0.2) | | ***-0.5***  ***(-0.9, -0.1)^**^*** | | -0.4  (-0.8, 0.03) | | -0.2  (-0.6, 0.2) | | 0.03  (-0.4, 0.5) | | ***-0.7***  ***(-0.9, -0.4)^***^*** | | ***-0.5***  ***(-0.8, -0.2)^**^*** | | |  |
| % sites PPD ≥ 5 mm | | ***-0.2***  ***(-0.3, -0.07)^**^*** | | -0.07  (-0.2, 0.06) | | ***-0.2***  ***(-0.3, -0.03)^*^*** | | -0.05  (-0.2, 0.1) | | -0.1  (-0.3, 0.05) | | -0.08  (-0.3, 0.1) | | -0.08  (-0.3, 0.1) | | 0.02  (-0.2, 0.2) | | ***-0.2***  ***(-0.4, -0.1)^**^*** | | ***-0.2***  ***(-0.3, -0.03)^*^*** | | |  |
| % sites PPD ≥ 6 mm | | ***-0.09***  ***(-0.2, -0.02)^*^*** | | -0.04  (-0.1, 0.03) | | ***-0.1***  ***(-0.2, -0.02)^*^*** | | -0.05  (-0.1, 0.04) | | -0.04  (-0.2, 0.06) | | -0.02  (-0.1, 0.1) | | -0.06  (-0.2, 0.06) | | -0.03  (-0.2, 0.1) | | ***-0.09***  ***(-0.2, -0.04)^**^*** | | ***-0.08***  ***(-0.1, -0.01)^*^*** | | |  |
| **CAL** | |  | |  | |  | |  | |  | |  | |  | |  | |  | |  | | |  |
| % sites CAL ≥ 3 mm | | ***-2.3***  ***(-3.1, -1.5)^***^*** | | ***-1.3***  ***(-2.1, -0.6)^**^*** | | ***-2.4***  ***(-3.2, -1.6)^***^*** | | ***-1.3***  ***(-2.0, -0.5)^**^*** | | ***-2.6***  ***(-3.9, -1.3)^***^*** | | ***-1.6***  ***(-2.8, -0.4)^**^*** | | ***-1.9***  ***(-3.1, -0.7)^**^*** | | ***-1.8***  ***(-3.1, -0.5)^**^*** | | ***-2.4***  ***(-3.2, -1.6)^***^*** | | ***-1.2***  ***(-2.0, -0.4)^**^*** | | |  |
| % sites CAL ≥ 4 mm | | ***-1.4***  ***(-1.9, -0.8)^***^*** | | ***-0.8***  ***(-1.3, -0.2)^**^*** | | ***-1.4***  ***(-1.9, -0.8)^***^*** | | ***-0.7***  ***(-1.3, -0.2)^*^*** | | ***-1.8***  ***(-2.9, -0.7)^**^*** | | -1.1  (-2.2, 0.05) | | ***-1.1***  ***(-2.1, -0.2)^*^*** | | -1.0  (-2.0, 0.01) | | ***-1.5***  ***(-2.1, -0.9)^***^*** | | ***-0.8***  ***(-1.4, -0.3)^**^*** | | |  |
| % sites CAL ≥ 5 mm | | ***-0.8***  ***(-1.2, -0.4)^***^*** | | ***-0.4***  ***(-0.8, -0.05)^*^*** | | ***-0.8***  ***(-1.2, -0.4)^***^*** | | ***-0.4***  ***(-0.8, -0.06)^*^*** | | ***-1.0***  ***(-1.9, -0.1)^*^*** | | -0.6  (-1.5, 0.3) | | -0.7  (-1.4, 0.05) | | -0.6  (-1.3, 0.1) | | ***-0.8***  ***(-1.2, -0.5)^***^*** | | ***-0.5***  ***(-0.9, -0.1)^*^*** | | |  |
| % sites CAL ≥ 6 mm | | ***-0.4***  ***(-0.7, -0.2)^**^*** | | -0.2  (-0.5, 0.03) | | ***-0.4***  ***(-0.7, -0.2)^**^*** | | -0.2  (-0.5, 0.08) | | -0.5  (-1.1, 0.1) | | -0.3  (-0.9, 0.3) | | -0.3  (-0.7, 0.1) | | -0.2  (-0.7, 0.2) | | ***-0.5***  ***(-0.7, -0.2)^***^*** | | ***-0.3***  ***(-0.6, -0.07)^*^*** | | |  |
| **UK Biobank** | | | | | | | | | | | | | | | | | | | | | |  |  |
| **Bleeding gums (vs. healthy)** | ***0.8***  ***(0.7, 0.8) ^***^*** | | ***0.8***  ***(0.7, 0.9) ^***^*** | | ***0.8***  ***(0.7, 0.8) ^***^*** | | ***0.8***  ***(0.7, 0.9) ^***^*** | | ***0.8***  ***(0.7, 0.9) ^***^*** | | ***0.8***  ***(0.7, 0.9) ^***^*** | | ***0.8***  ***(0.7, 0.8) ^***^*** | | ***0.8***  ***(0.7, 0.9) ^***^*** | | ***0.8***  ***(0.7, 0.9) ^***^*** | | ***0.9***  ***(0.8, 0.9) ^***^*** | |  |  |  |
| **Painful gums (vs. healthy)** | ***0.6***  ***(0.5, 0.6) ^***^*** | | ***0.7***  ***(0.6, 0.7) ^***^*** | | ***0.5***  ***(0.4, 0.6) ^***^*** | | ***0.6***  ***(0.5, 0.7) ^***^*** | | ***0.6***  ***(0.5, 0.7) ^***^*** | | ***0.7***  ***(0.6, 0.8) ^***^*** | | ***0.5***  ***(0.4, 0.6) ^***^*** | | ***0.6***  ***(0.5, 0.7) ^***^*** | | ***0.6***  ***(0.5, 0.6) ^***^*** | | ***0.7***  ***(0.6, 0.8) ^***^*** | |  |  |  |
| **Loose teeth (vs. healthy)** | ***0.7***  ***(0.6, 0.7) ^***^*** | | ***0.8***  ***(0.7, 0.9) ^**^*** | | ***0.6***  ***(0.5, 0.7) ^***^*** | | ***0.7***  ***(0.7, 0.9) ^***^*** | | ***0.8***  ***(0.7, 0.9) ^**^*** | | 0.9  (0.8, 1.1) | | ***0.7***  ***(0.6, 0.8) ^***^*** | | ***0.8***  ***(0.7, 0.9) ^*^*** | | ***0.7***  ***(0.6, 0.8) ^***^*** | | ***0.8***  ***(0.7, 0.9) ^*^*** | |  |  |  |
| **Any periodontal disease (vs. healthy)** | ***0.8***  ***(0.6, 0.9) ^**^*** | | ***0.7***  ***(0.6, 0.9) ^*^*** | | ***0.6***  ***(0.5, 0.8) ^**^*** | | ***0.7***  ***(0.7, 0.9) ^***^*** | | ***0.7***  ***(0.6, 0.9) ^**^*** | | 0.9  (0.8, 1.0) | | ***0.6***  ***(0.5, 0.9) ^**^*** | | ***0.7***  ***(0.5, 0.9) ^*^*** | | ***0.7***  ***(0.6, 0.8) ^***^*** | | ***0.8***  ***(0.6, 0.9) ^*^*** | |  |  |  |

Abbreviations. CAL, clinical attachment level; CI, confidence interval; MD, difference in means; mm, millimeter; OR, odds ratio; PPD, probing pocket depth; %, percentage.

^*^ p<0.05; ^**^ p<0.01; ^***^ p<0.001.

**Table S9**. Association between categories of healthy lifestyle score (2-3 vs. 0-1 healthy lifestyles) and periodontitis in systemically healthy individuals only (sensitivity analyses) overall and by subgroups of age and sex.

| **2-3 healthy lifestyles (vs. 0-1 healthy lifestyles) as exposure – OR/MD (95% CI)** | | | | | | | | | | | | | | | | | | | | | |
| --- | --- | --- | --- | --- | --- | --- | --- | --- | --- | --- | --- | --- | --- | --- | --- | --- | --- | --- | --- | --- | --- |
| **Outcomes** | | **Overall** | | | | **Age** | | | | | | | | **Sex** | | | | | | | |
|  |  |  |  |  |  | **< 60 years** | | | | **≥ 60 years** | | | | **Males** | | | **Females** | | | | |
|  | | ***Crude*** | | ***Adjusted*** | | ***Crude*** | | ***Adjusted*** | | ***Crude*** | | ***Adjusted*** | | ***Crude*** | | ***Adjusted*** | ***Crude*** | | ***Adjusted*** | |  |
| **NHANES** | | | | | | | | | | | | | | | | | | | | |  |
| **Periodontitis** | | ***0.5***  ***(0.4, 0.7)^***^*** | | ***0.5***  ***(0.3, 0.7) ^***^*** | | ***0.5***  ***(0.4, 0.6)^***^*** | | ***0.5***  ***(0.3, 0.7) ^***^*** | | ***0.4***  ***(0.3, 0.6)^**^*** | | ***0.5***  ***(0.3, 0.6) ^***^*** | | ***0.5***  ***(0.4, 0.8)^***^*** | | ***0.5***  ***(0.3, 0.8) ^**^*** | ***0.5***  ***(0.3, 0.7) ^***^*** | | ***0.4***  ***(0.3, 0.7) ^**^*** | |  |
| **Periodontitis severity** | |  | |  | |  | |  | |  | |  | |  | |  |  | |  | |  |
| *Mild periodontitis* | | ***0.3***  ***(0.2, 0.6)^***^*** | | 0.5  (0.2, 1.0) | | ***0.3***  ***(0.2, 0.5)^***^*** | | ***0.5***  ***(0.2, 0.9) ^*^*** | | NE | | NE | | 0.5  (0.2, 1.1) | | 0.7  (0.3, 1.2) | ***0.2***  ***(0.09, 0.6) ^***^*** | | 0.3  (0.09, 1.0) | |  |
| *Moderate periodontitis* | | ***0.4***  ***(0.3, 0.5)^***^*** | | ***0.4***  ***(0.3, 0.6) ^***^*** | | ***0.3***  ***(0.2, 0.4)^***^*** | | ***0.4***  ***(0.3, 0.7) ^**^*** | | ***0.3***  ***(0.2, 0.6)^***^*** | | ***0.4***  ***(0.3, 0.8) ^***^*** | | ***0.3***  ***(0.2, 0.6) ^***^*** | | ***0.4***  ***(0.3, 0.6) ^***^*** | ***0.4***  ***(0.3, 0.6) ^***^*** | | ***0.5***  ***(0.3, 0.8) ^*^*** | |  |
| *Severe periodontitis* | | ***0.1***  ***(0.08, 0.3)^***^*** | | ***0.2***  ***(0.08, 0.4) ^***^*** | | ***0.1***  ***(0.07, 0.2)^***^*** | | ***0.2***  ***(0.07, 0.5) ^**^*** | | ***0.1***  ***(0.03, 0.2)^**^*** | | ***0.1***  ***(0.09, 0.3) ^***^*** | | ***0.1***  ***(0.05, 0.2) ^***^*** | | ***0.1***  ***(0.03, 0.5) ^***^*** | ***0.3 (0.09, 0.7) ^**^*** | | ***0.2***  ***(0.06, 0.9) ^*^*** | |  |
| **PPD** | |  | |  | |  | |  | |  | |  | |  | |  |  | |  | |  |
| % sites PPD ≥ 4 mm | | ***-1.8***  ***(-2.5, -1.1)^***^*** | | ***-1.3***  ***(-1.9, -0.6) ^***^*** | | ***-1.9***  ***(-2.6, -1.1)^***^*** | | ***-1.4***  ***(-2.2, -0.6) ^**^*** | | -0.4  (-0.5, 1.0) | | -0.1  (-0.3, 0.2) | | ***-2.1***  ***(-3.4, -0.9) ^**^*** | | ***-1.6***  ***(-2.9, -0.3) ^*^*** | ***-1.2***  ***(-2.2, -0.3) ^*^*** | | -0.9  (-1.9, 0.04) | |  |
| % sites PPD ≥ 5 mm | | ***-0.5***  ***(-0.8, -0.1)^*^*** | | -0.3  (-0.7, 0.04) | | ***-0.5***  ***(-0.9, -0.09)^*^*** | | -0.4  (-0.8, 0.04) | | 0.2  (-0.1, 0.2) | | 0.2  (-0.2, 0.4) | | -0.7  (-1.3, 0.03) | | -0.5  (-1.2, 0.2) | -0.2  (-0.5, 0.09) | | -0.1  (-0.5, 0.2) | |  |
| % sites PPD ≥ 6 mm | | -0.1  (-0.4, 0.08) | | -0.09  (-0.3, 0.1) | | -0.1  (-0.4, 0.09) | | -0.09  (-0.4, 0.2) | | 0.05  (-0.1, 0.3) | | 0.06  (-0.2, 0.3) | | -0.2  (-0.6, 0.2) | | -0.1  (-0.6, 0.3) | -0.02  (-0.1, 0.09) | | -0.003  (-0.1, 0.1) | |  |
| **CAL** | |  | |  | |  | |  | |  | |  | |  | |  |  | |  | |  |
| % sites CAL ≥ 3 mm | | ***-5.8***  ***(-8.7, -2.9)^***^*** | | ***-5.5***  ***(-7.9, -3.0) ^***^*** | | ***-5.8***  ***(-8.4, -3.2)^***^*** | | ***-5.1***  ***(-7.6, -2.7) ^***^*** | | ***-11.1***  ***(-16.2, -3.1)^***^*** | | ***-9.4***  ***(-13.8, -2.9) ^***^*** | | ***-7.1***  ***(-10.9, -3.2) ^**^*** | | ***-7.1***  ***(-11.1, -3.1) ^**^*** | ***-4.0***  ***(-6.6, -1.5) ^**^*** | | ***-3.9***  ***(-6.1, -1.6) ^**^*** | |  |
| % sites CAL ≥ 4 mm | | ***-3.9***  ***(-6.1, -1.9)^***^*** | | ***-3.9***  ***(-5.9, -1.9) ^***^*** | | ***-4.0***  ***(-6.1, -1.9)^***^*** | | ***-3.7***  ***(-5.8, -1.6) ^***^*** | | ***-6.4***  ***(-9.1, -1.3)^***^*** | | ***-5.9***  ***(-8.7, -1.9) ^***^*** | | ***-5.1***  ***(-7.8, -2.4) ^***^*** | | ***-5.1***  ***(-8.1, 2.1) ^**^*** | ***-2.6***  ***(-4.6, -0.6) ^*^*** | | ***-2.6***  ***(-4.4, -0.7) ^**^*** | |  |
| % sites CAL ≥ 5 mm | | ***-2.3***  ***(-3.7, -0.9)^**^*** | | ***-2.2***  ***(-3.6, -0.9) ^**^*** | | ***-2.4***  ***(-3.9, -0.9)^**^*** | | ***-2.3***  ***(-3.8, -0.7) ^**^*** | | ***-2.4***  ***(-3.3, -1.0)^**^*** | | ***-2.2***  ***(-3.5, -1.2) ^**^*** | | ***-3.3***  ***(-5.3, -1.3) ^**^*** | | ***-3.3***  ***(-5.4, -1.1) ^**^*** | ***-1.2***  ***(-2.1, -0.2) ^*^*** | | ***-1.1***  ***(-2.0, -0.3) ^*^*** | |  |
| % sites CAL ≥ 6 mm | | ***-1.4***  ***(-2.5, -0.4)^**^*** | | ***-1.4***  ***(-2.5, -0.3) ^*^*** | | ***-1.5***  ***(-2.6, -0.4)^*^*** | | ***-1.4***  ***(-2.6, -0.3) ^*^*** | | ***-1.0***  ***(-2.1, -0.3)^*^*** | | ***-0.9***  ***(-2.0, -0.4) ^**^*** | | ***-2.3***  ***(-3.9, -0.7) ^**^*** | | ***-2.2***  ***(-3.9, -0.5) ^*^*** | -0.5  (-0.9, 0.02) | | ***-0.5***  ***(-0.9, -0.05) ^*^*** | |  |
| **UK Biobank** | | | | | | | | | | | | | | | | | | | |  |  |
| **Bleeding gums (vs. healthy)** | 0.9  (0.8, 1.0) | | 0.9  (0.8, 1.0) | | 0.9  (0.8, 1.1) | | 0.9  (0.8, 1.1) | | 0.9  (0.6, 1.1) | | 0.8  (0.6, 1.1) | | 0.9  (0.8, 1.2) | | 0.9  (0.8, 1.1) | | ***0.9***  ***(0.7, 0.9) ^*^*** | 0.9  (0.7, 1.0) | |  |  |
| **Painful gums (vs. healthy)** | ***0.7***  ***(0.6, 0.9) ^*^*** | | ***0.7***  ***(0.5, 0.9) ^**^*** | | ***0.7***  ***(0.5, 0.9) ^*^*** | | ***0.7***  ***(0.5, 0.9) ^**^*** | | 0.9  (0.5, 1.5) | | 0.8  (0.4, 1.4) | | 0.8  (0.6, 1.5) | | 0.8  (0.5, 1.1) | | ***0.7***  ***(0.5, 0.9) ^*^*** | ***0.6***  ***(0.5, 0.9) ^**^*** | |  |  |
| **Loose teeth (vs. healthy)** | ***0.7***  ***(0.6, 0.9) ^*^*** | | ***0.7***  ***(0.5, 0.9) ^**^*** | | 0.7  (0.5, 1.0) | | ***0.7***  ***(0.5, 0.9) ^*^*** | | 0.7  (0.4, 1.1) | | 0.6  (0.4, 1.1) | | ***0.9***  ***(0.7, 0.9) ^*^*** | | 0.9  (0.6, 1.4) | | ***0.6***  ***(0.4, 0.8) ^**^*** | ***0.5***  ***(0.4, 0.8) ^***^*** | |  |  |
| **Any periodontal disease (vs. healthy)** | ***0.8***  ***(0.7, 0.9) ^*^*** | | ***0.6***  ***(0.5, 0.8) ^**^*** | | ***0.8***  ***(0.5, 0.9) ^*^*** | | ***0.8***  ***(0.6, 0.9) ^**^*** | | 0.9  (0.5, 1.52 | | 0.9  (0.6, 1.3) | | ***0.8***  ***(0.7, 0.9) ^*^*** | | 0.9  (0.7, 1.2) | | ***0.8***  ***(0.6, 0.9) ^*^*** | ***0.7***  ***(0.5, 0.9) ^*^*** | |  |  |

Abbreviations. CAL, clinical attachment level; CI, confidence interval; MD, difference in means; mm, millimeter; OR, odds ratio; PPD, probing pocket depth; %, percentage.

^*^ p<0.05; ^**^ p<0.01; ^***^ p<0.001.

**Table S10**. Association between categories of healthy lifestyle score (4-5 vs. 0-1 healthy lifestyles) and periodontitis in systemically healthy individuals only (sensitivity analyses) overall and by subgroups of age and sex.

| **4-5 healthy lifestyles (vs. 0-1 healthy lifestyles) as exposure – OR/MD (95% CI)** | | | | | | | | | | | | | | | | | | | | | |
| --- | --- | --- | --- | --- | --- | --- | --- | --- | --- | --- | --- | --- | --- | --- | --- | --- | --- | --- | --- | --- | --- |
| **Outcomes** | | **Overall** | | | | **Age** | | | | | | | | **Sex** | | | | | | | |
|  |  |  |  |  |  | **< 60 years** | | | | **≥ 60 years** | | | | **Males** | | | **Females** | | | | |
|  | | ***Crude*** | | ***Adjusted*** | | ***Crude*** | | ***Adjusted*** | | ***Crude*** | | ***Adjusted*** | | ***Crude*** | | ***Adjusted*** | ***Crude*** | | ***Adjusted*** | |  |
| **NHANES** | | | | | | | | | | | | | | | | | | | | |  |
| **Periodontitis** | | ***0.3***  ***(0.2, 0.4)^***^*** | | ***0.3***  ***(0.2, 0.5) ^***^*** | | ***0.3***  ***(0.2, 0.3)^***^*** | | ***0.3***  ***(0.2, 0.5) ^***^*** | | ***0.3***  ***(0.1, 0.5)^**^*** | | ***0.4***  ***(0.2, 0.6) ^**^*** | | ***0.3***  ***(0.2, 0.4) ^***^*** | | ***0.3***  ***(0.2, 0.5) ^***^*** | ***0.4***  ***(0.2, 0.5) ^***^*** | | ***0.4***  ***(0.2, 0.6) ^***^*** | |  |
| **Periodontitis severity** | |  | |  | |  | |  | |  | |  | |  | |  |  | |  | |  |
| *Mild periodontitis* | | ***0.5***  ***(0.3, 0.6)^***^*** | | ***0.5***  ***(0.4, 0.7) ^***^*** | | ***0.3***  ***(0.2, 0.5)^***^*** | | ***0.5***  ***(0.3, 0.7) ^***^*** | | NE | | NE | | 0.5  (0.2, 1.1) | | ***0.5***  ***(0.3, 0.7) ^**^*** | ***0.2***  ***(0.09, 0.6) ^**^*** | | 0.7  (0.4, 1.1) | |  |
| *Moderate periodontitis* | | ***0.4***  ***(0.3, 0.5)^***^*** | | ***0.4***  ***(0.3, 0.5) ^***^*** | | ***0.3***  ***(0.2, 0.4)^***^*** | | ***0.3***  ***(0.2, 0.4) ^***^*** | | ***0.3***  ***(0.1, 0.5)^***^*** | | ***0.4***  ***(0.3, 0.8) ^**^*** | | ***0.3***  ***(0.2, 0.6) ^***^*** | | ***0.4***  ***(0.3, 0.5) ^***^*** | ***0.4***  ***(0.3, 0.6) ^***^*** | | ***0.5***  ***(0.4, 0.6) ^***^*** | |  |
| *Severe periodontitis* | | ***0.2***  ***(0.1, 0.2)^***^*** | | ***0.2***  ***(0.1, 0.2) ^***^*** | | ***0.1***  ***(0.07, 0.2)^***^*** | | ***0.2***  ***(0.1, 0.2) ^***^*** | | ***0.1***  ***(0.05, 0.3)^***^*** | | ***0.2***  ***(0.09, 0.3) ^***^*** | | ***0.1***  ***(0.05, 0.2) ^***^*** | | ***0.2***  ***(0.1, 0.3) ^***^*** | ***0.3***  ***(0.09, 0.7) ^**^*** | | ***0.2***  ***(0.1, 0.3) ^***^*** | |  |
| **PPD** | |  | |  | |  | |  | |  | |  | |  | |  |  | |  | |  |
| % sites PPD ≥ 4 mm | | ***-2.8***  ***(-3.4, -2.1)^***^*** | | ***-1.7***  ***(-2.4, -1.0) ^***^*** | | ***-2.9***  ***(-3.6, -2.2)^***^*** | | ***-1.8***  ***(-2.6, -1.1) ^***^*** | | ***-1.0***  ***(-2.9, -0.1)^**^*** | | -0.6  (-1.2, 0.2) | | ***-3.6***  ***(-4.8, -2.3) ^***^*** | | ***-2.1***  ***(-3.3, -0.8) ^**^*** | ***-1.8***  ***(-2.7, -0.9) ^***^*** | | ***-1.3***  ***(-2.2, -0.3) ^*^*** | |  |
| % sites PPD ≥ 5 mm | | ***-0.8***  ***(-1.1, -0.4)^***^*** | | ***-0.5***  ***(-0.8, -0.1) ^**^*** | | ***-0.8***  ***(-1.2, -0.4)^***^*** | | ***-0.5***  ***(-0.9, -0.1) ^*^*** | | -0.08  (-0.9, 1.0) | | -0.04  (-0.2, 0.9) | | ***-1.1***  ***(-1.7, -0.4) ^**^*** | | -0.6  (-1.3, 0.05) | ***-0.4***  ***(-0.7, -0.1) ^**^*** | | -0.3  (-0.6, 0.02) | |  |
| % sites PPD ≥ 6 mm | | ***-0.3***  ***(-0.5, -0.07)^**^*** | | -0.2  (-0.4, 0.04) | | ***-0.3***  ***(-0.5, -0.07)^*^*** | | -0.2  (-0.4, 0.06) | | -0.02  (-0.1, 0.3) | | -0.02  (-0.1, 0.4) | | ***-0.4***  ***(-0.8, -0.03) ^*^*** | | -0.2  (-0.6, 0.2) | ***-0.09***  ***(-0.2, -0.003) ^*^*** | | -0.06  (-0.2, 0.04) | |  |
| **CAL** | |  | |  | |  | |  | |  | |  | |  | |  |  | |  | |  |
| % sites CAL ≥ 3 mm | | ***-8.4***  ***(-11.2, -5.6)^***^*** | | ***-7.2***  ***(-9.9, -4.4) ^***^*** | | ***-8.5***  ***(-11.1, -5.9)^***^*** | | ***-6.5***  ***(-9.2, -3.8) ^***^*** | | ***-14.7***  ***(-18.1, -6.9)^**^*** | | ***-12.6***  ***(-21.1, -5.3) ^***^*** | | ***-10.5***  ***(-14.6, -6.4) ^***^*** | | ***-9.0***  ***(-13.7, -4.3) ^***^*** | ***-5.8***  ***(-8.3, -3.3) ^***^*** | | ***-5.3***  ***(-7.8, -2.9) ^***^*** | |  |
| % sites CAL ≥ 4 mm | | ***-5.5***  ***(-7.5, -3.4)^***^*** | | ***-4.9***  ***(-6.9, -2.8) ^***^*** | | ***-5.4***  ***(-7.5, -3.4)^***^*** | | ***-4.4***  ***(-6.6, -2.3) ^***^*** | | ***-9.1***  ***(-10.8, -4.2)^***^*** | | ***-8.1***  ***(-10.1, -3.9) ^***^*** | | ***-7.0***  ***(-9.7, -4.3) ^***^*** | | ***-6.2***  ***(-9.5, -2.9) ^***^*** | ***-3.7***  ***(-5.6, -1.7) ^***^*** | | ***-3.5***  ***(-5.2, -1.7) ^***^*** | |  |
| % sites CAL ≥ 5 mm | | ***-3.1***  ***(-4.4, -1.7)^***^*** | | ***-2.7***  ***(-4.1, -1.3) ^***^*** | | ***-3.1***  ***(-4.5, -1.7)^***^*** | | ***-2.6***  ***(-4.1, -1.1) ^**^*** | | ***-3.8***  ***(-4.1, -1.9)^***^*** | | ***-3.3***  ***(-3.9, -1.3) ^***^*** | | ***-4.3***  ***(-6.2, -2.4) ^***^*** | | ***-3.8***  ***(-6.0, -1.6) ^**^*** | ***-1.7***  ***(-2.6, -0.7) ^**^*** | | ***-1.6***  ***(-2.5, -0.7) ^**^*** | |  |
| % sites CAL ≥ 6 mm | | ***-1.8***  ***(-2.8, -0.8)^**^*** | | ***-1.6***  ***(-2.7, -0.6) ^**^*** | | ***-1.9***  ***(-2.9, -0.8)^**^*** | | ***-1.6***  ***(-2.7, -0.5) ^**^*** | | ***-1.7***  ***(-2.8, -0.7)^**^*** | | ***-1.5***  ***(-2.5, -0.4) ^**^*** | | ***-2.9***  ***(-4.4, -1.3) ^**^*** | | ***-2.5***  ***(-4.2, -0.7) ^**^*** | ***-0.7***  ***(-1.2, -0.3) ^**^*** | | ***-0.7***  ***(-1.2, -0.3) ^**^*** | |  |
| **UK Biobank** | | | | | | | | | | | | | | | | | | | |  |  |
| **Bleeding gums (vs. healthy)** | ***0.8***  ***(0.7, 0.9) ^**^*** | | ***0.8***  ***(0.7, 0.9) ^***^*** | | ***0.8***  ***(0.7, 0.9) ^*^*** | | ***0.8***  ***(0.7, 0.9) ^**^*** | | 0.8  (0.6, 1.0) | | ***0.7***  ***(0.5, 0.9) ^*^*** | | ***0.7***  ***(0.6, 0.9) ^**^*** | | ***0.7***  ***(0.6, 0.9) ^**^*** | | ***0.8***  ***(0.7, 0.9) ^**^*** | ***0.8***  ***(0.7, 0.9) ^**^*** | |  |  |
| **Painful gums (vs. healthy)** | ***0.7***  ***(0.6, 0.9) ^*^*** | | ***0.6***  ***(0.5, 0.8) ^***^*** | | ***0.6***  ***(0.5, 0.8) ^**^*** | | ***0.5***  ***(0.4, 0.7) ^***^*** | | 1.0  (0.6, 1.8) | | 0.9  (0.5, 1.6) | | 0.8  (0.5, 1.2) | | 0.6  (0.4, 1.0) | | ***0.6***  ***(0.5, 0.9) ^**^*** | ***0.6***  ***(0.4, 0.8) ^***^*** | |  |  |
| **Loose teeth (vs. healthy)** | ***0.7***  ***(0.5, 0.9) ^*^*** | | ***0.5***  ***(0.4, 0.7) ^***^*** | | ***0.7***  ***(0.5, 0.9) ^*^*** | | ***0.5***  ***(0.3, 0.7) ^***^*** | | 0.7  (0.4, 1.1) | | 0.6  (0.4, 1.1) | | 0.9  (0.6, 1.4) | | 0.7  (0.4, 1.1) | | ***0.6***  ***(0.4, 0.8) ^**^*** | ***0.4***  ***(0.3, 0.6) ^***^*** | |  |  |
| **Any periodontal disease (vs. healthy)** | ***0.9***  ***(0.6, 0.9) ^*^*** | | ***0.8***  ***(0.7, 0.9)^**^*** | | ***0.7***  ***(0.6, 0.9) ^*^*** | | ***0.8***  ***(0.7, 0.9) ^**^*** | | 0.9  (0.6, 1.1) | | ***0.8***  ***(0.6, 0.9) ^*^*** | | ***0.8***  ***(0.6, 0.9) ^**^*** | | ***0.8***  ***(0.7, 0.9) ^**^*** | | ***0.7***  ***(0.5, 0.9) ^**^*** | ***0.6***  ***(0.4, 0.8) ^**^*** | |  |  |

Abbreviations. CAL, clinical attachment level; CI, confidence interval; MD, difference in means; mm, millimeter; OR, odds ratio; PPD, probing pocket depth; %, percentage.

^*^ p<0.05; ^**^ p<0.01; ^***^ p<0.001.

**Table S11**. Association between categories of healthy lifestyle score (2-3 vs. 0-1 healthy lifestyles) and periodontitis across subgroups of BMI (sensitivity analyses).

| **2-3 healthy lifestyles (vs. 0-1 healthy lifestyles) as exposure – OR/MD (95% CI)** | | | | | | | | |
| --- | --- | --- | --- | --- | --- | --- | --- | --- |
| **Outcomes** | **Underweight** | | **Normal weight** | | **Overweight** | | **Obese** | |
|  | ***Crude*** | ***Adjusted*** | ***Crude*** | ***Adjusted*** | ***Crude*** | ***Crude*** | ***Adjusted*** | ***Crude*** |
| **NHANES** | | | | | | | | |
| **Periodontitis** | 0.2 (NE) | 0.2 (NE) | ***0.3***  ***(0.3, 0.4) ^***^*** | ***0.3***  ***(0.2, 0.5) ^***^*** | ***0.5***  ***(0.4, 0.7) ^***^*** | ***0.5***  ***(0.3, 0.7) ^***^*** | ***0.6***  ***(0.5, 0.7) ^***^*** | ***0.5***  ***(0.4, 0.7) ^***^*** |
| **Periodontitis severity** |  |  |  |  |  |  |  |  |
| *Mild periodontitis* | NE | NE | ***0.3***  ***(0.1, 0.8) ^*^*** | ***0.3***  ***(0.1, 0.7) ^**^*** | 0.7  (0.4, 1.2) | ***0.9***  ***(0.4, 2.0)*** | ***0.4***  ***(0.2, 0.7) ^**^*** | ***0.4***  ***(0.2, 0.8) ^*^*** |
| *Moderate periodontitis* | 0.02 (NE) | 0.05 (NE) | ***0.4***  ***(0.3, 0.5) ^***^*** | ***0.1***  ***(0.07, 0.2) ^***^*** | ***0.5***  ***(0.4, 0.7) ^***^*** | ***0.4***  ***(0.3, 0.7) ^***^*** | ***0.4***  ***(0.3, 0.6) ^***^*** | ***0.3***  ***(0.2, 0.5) ^***^*** |
| *Severe periodontitis* | 0.004 (NE) | 0.003 (NE) | ***0.1***  ***(0.04, 0.1) ^***^*** | ***0.04***  ***(0.02, 0.09) ^***^*** | ***0.1***  ***(0.09, 0.2) ^***^*** | ***0.09***  ***(0.05, 0.2) ^***^*** | ***0.4***  ***(0.2, 0.6) ^***^*** | ***0.3***  ***(0.2, 0.6) ^***^*** |
| **PPD** |  |  |  |  |  |  |  |  |
| % sites PPD ≥ 4 mm | -6.4 (NE) | -6.9 (NE) | ***-3.1***  ***(-4.2, -1.9) ^***^*** | ***-2.1***  ***(-3.2, -0.9) ^**^*** | ***-2.4***  ***(-3.2, -1.6) ^***^*** | ***-2.1***  ***(-2.9, -1.1) ^***^*** | ***-1.0***  ***(-1.9, -0.09) ^**^*** | -0.7  (-1.6, 0.3) |
| % sites PPD ≥ 5 mm | -1.9 (NE) | -2.3 (NE) | ***-0.9***  ***(-1.4, -0.4) ^**^*** | ***-0.6***  ***(-1.0, -0.09) ^*^*** | ***-1.0***  ***(-1.5, -0.5) ^***^*** | ***-0.9***  ***(-1.4, -0.4) ^**^*** | -0.2  (-0.7, 0.2) | -0.2  (-0.6, 0.3) |
| % sites PPD ≥ 6 mm | -0.9 (NE) | -0.4 (NE) | ***-0.3***  ***(-0.6, -0.04) ^*^*** | -0.1  (-0.3, 0.09) | ***-0.4***  ***(-0.6, -0.1) ^**^*** | ***-0.3***  ***(-0.6, -0.09) ^**^*** | -0.03  (-0.2, 0.2) | -0.01  (-0.2, 0.2) |
| **CAL** |  |  |  |  |  |  |  |  |
| % sites CAL ≥ 3 mm | -23.3 (NE) | -15.2 (NE) | ***-10.4***  ***(-13.0, -7.7) ^***^*** | ***-9.1***  ***(-12.1, -6.2) ^***^*** | ***-6.7***  ***(-8.7, -4.6) ^***^*** | ***-6.9***  ***(-8.9, -4.9) ^***^*** | ***-3.9***  ***(-5.6, -2.2) ^***^*** | ***-3.9***  ***(-5.5, -2.4) ^***^*** |
| % sites CAL ≥ 4 mm | -23.9 (NE) | -21.2 (NE) | ***-6.9***  ***(-8.8, -4.2) ^***^*** | ***-5.7***  ***(-7.9, -3.6) ^***^*** | ***-4.7***  ***(-6.2, -3.2) ^***^*** | ***-5.1***  ***(-6.5, -3.6) ^***^*** | ***-2.2***  ***(-3.4, -1.1) ^***^*** | ***-2.3***  ***(-3.5, -1.1) ^***^*** |
| % sites CAL ≥ 5 mm | -17.8 (NE) | -13.9 (NE) | ***-3.9***  ***(-5.2, -2.7) ^***^*** | ***-3.2***  ***(-4.5, -1.9) ^***^*** | ***-2.9***  ***(-3.9, -1.9) ^***^*** | ***-3.2***  ***(-4.2, -2.2) ^***^*** | ***-1.0***  ***(-1.8, -0.3) ^**^*** | ***-1.0***  ***(-1.8, -0.2) ^*^*** |
| % sites CAL ≥ 6 mm | -14.6 (NE) | -6.1 (NE) | ***-2.0***  ***(-2.9, -1.2) ^***^*** | ***-1.5***  ***(-2.4, -0.5) ^**^*** | ***-1.8***  ***(-2.4, -1.1) ^***^*** | ***-1.8***  ***(-2.4, -1.1) ^***^*** | ***-0.7***  ***(-1.2, -0.1) ^*^*** | ***-0.6***  ***(-1.2, -0.008) ^*^*** |
| **UK Biobank** |  |  |  |  |  |  |  |  |
| **Bleeding gums (vs. healthy)** | 0.9  (0.8, 1.0) | 0.9  (0.8, 1.0) | 0.5  (0.2, 1.6) | 0.4  (0.1, 1.1) | ***0.8***  ***(0.7, 0.9) ^**^*** | ***0.8***  ***(0.7, 0.9) ^***^*** | 1.2  (0.9, 1.4) | 1.1  (0.9, 1.3) |
| **Painful gums (vs. healthy)** | 0.8  (0.6, 1.1) | 0.8  (0.6, 1.1) | 0.5  (0.09, 2.3) | 0.5  (0.1, 2.8) | ***0.8***  ***(0.6, 0.9) ^*^*** | ***0.7***  ***(0.6, 0.9) ^*^*** | 1.4  (0.9, 2.1) | 1.1  (0.8, 1.7) |
| **Loose teeth (vs. healthy)** | ***0.6***  ***(0.4, 0.7) ^***^*** | ***0.5***  ***(0.4, 0.7) ^***^*** | 0.8  (0.1, 6.6) | 0.8  (0.1, 7.2) | 0.8  (0.6, 1.1) | ***0.7***  ***(0.5, 0.9) ^*^*** | 0.9  (0.7, 1.3) | 0.8  (0.5, 1.1) |
| **Any periodontal disease (vs. healthy)** | ***0.7***  ***(0.4, 0.9)^*^*** | ***0.6***  ***(0.3, 0.8) ^***^*** | 0.7  (0.3, 2.4) | 0.6  (0.3, 5.1) | 0.9  (0.4, 1.3) | ***0.6***  ***(0.3, 0.9) ^*^*** | 1.0  (0.7, 1.2) | 0.9  (0.4, 1.3) |

Abbreviations. CAL, clinical attachment level; CI, confidence interval; MD, difference in means; mm, millimeter; NE, not estimable; OR, odds ratio; PPD, probing pocket depth; %, percentage.

^*^ p<0.05; ^**^ p<0.01; ^***^ p<0.001.

**Table S12**. Association between categories of healthy lifestyle score (4-5 vs. 0-1 healthy lifestyles) and periodontitis across subgroups of BMI (sensitivity analyses).

| **4-5 healthy lifestyles (vs. 0-1 healthy lifestyles) as exposure – OR/MD (95% CI)** | | | | | | | | |
| --- | --- | --- | --- | --- | --- | --- | --- | --- |
| **Outcomes** | **Underweight** | | **Normal weight** | | **Overweight** | | **Obese** | |
|  | ***Crude*** | ***Adjusted*** | ***Crude*** | ***Adjusted*** | ***Crude*** | ***Adjusted*** | ***Crude*** | ***Adjusted*** |
| **NHANES** |  |  |  |  |  |  |  |  |
| **Periodontitis** | 0.01 (NE) | 0.01 (NE) | ***0.2***  ***(0.1, 0.3) ^***^*** | ***0.2***  ***(0.1, 0.3) ^***^*** | ***0.4***  ***(0.3, 0.5) ^***^*** | ***0.4***  ***(0.3, 0.5) ^***^*** | ***0.4***  ***(0.3, 0.6) ^***^*** | ***0.4***  ***(0.3, 0.5) ^***^*** |
| **Periodontitis severity** |  |  |  |  |  |  |  |  |
| *Mild periodontitis* | ***0.5***  ***(0.3, 0.6) ^***^*** | ***0.7***  ***(0.5, 0.9) ^*^*** | ***0.5***  ***(0.3, 0.6) ^***^*** | ***0.5***  ***(0.3, 0.8) ^**^*** | ***0.5***  ***(0.3, 0.6) ^***^*** | ***0.5***  ***(0.3, 0.8) ^***^*** | ***0.5***  ***(0.3, 0.6) ^***^*** | ***0.5***  ***(0.3, 0.8) ^**^*** |
| *Moderate periodontitis* | ***0.4***  ***(0.3, 0.5) ^**^*** | ***0.6***  ***(0.2, 0.8) ^**^*** | ***0.4***  ***(0.3, 0.5) ^***^*** | ***0.3***  ***(0.2, 0.4) ^***^*** | ***0.4***  ***(0.3, 0.5) ^***^*** | ***0.3***  ***(0.2, 0.4) ^***^*** | ***0.4***  ***(0.3, 0.5) ^***^*** | ***0.3***  ***(0.2, 0.4) ^***^*** |
| *Severe periodontitis* | ***0.2***  ***(0.1, 0.2) ^**^*** | ***0.3***  ***(0.1, 0.4) ^***^*** | ***0.2***  ***(0.1, 0.2) ^***^*** | ***0.1***  ***(0.08, 0.2) ^***^*** | ***0.2***  ***(0.1, 0.2) ^***^*** | ***0.1***  ***(0.08, 0.2) ^***^*** | ***0.2***  ***(0.1, 0.2) ^***^*** | ***0.1***  ***(0.09, 0.2) ^***^*** |
| **PPD** |  |  |  |  |  |  |  |  |
| % sites PPD ≥ 4 mm | -8.2 (NE) | -6.5 (NE) | ***-4.5***  ***(-5.6, -3.3) ^***^*** | ***-2.9***  ***(-4.1, -1.7) ^***^*** | ***-3.2***  ***(-3.9, -2.5) ^***^*** | ***-2.5***  ***(-3.3, -1.7) ^***^*** | ***-1.9***  ***(-2.7, -1.1) ^***^*** | ***-1.4***  ***(-2.3, -0.5) ^**^*** |
| % sites PPD ≥ 5 mm | -2.4 (NE) | -3.1 (NE) | ***-1.4***  ***(-1.9, -0.9) ^***^*** | ***-0.8***  ***(-1.3, -0.4) ^**^*** | ***-1.2***  ***(-1.6, -0.8) ^***^*** | ***-1.0***  ***(-1.5, -0.5) ^***^*** | ***-0.5***  ***(-0.9, -0.1) ^*^*** | -0.4  (-0.9, 0.07) |
| % sites PPD ≥ 6 mm | -1.2 (NE) | -0.5 (NE) | ***-0.5***  ***(-0.7, -0.2) ^**^*** | -0.2  (-0.4, 0.02) | ***-0.4***  ***(-0.6, -0.2) ^***^*** | ***-0.3***  ***(-0.6, -0.1) ^**^*** | -0.1  (-0.3, 0.07) | -0.09  (-0.3, 0.2) |
| **CAL** |  |  |  |  |  |  |  |  |
| % sites CAL ≥ 3 mm | -35.2 (NE) | -23.1 (NE) | ***-14.8***  ***(-17.6, -11.9) ^***^*** | ***-12.4***  ***(-15.7, -9.1) ^***^*** | ***-8.8***  ***(-10.9, -6.7) ^***^*** | ***-8.7***  ***(-10.9, -6.6) ^***^*** | ***-5.9***  ***(-7.4, -4.4) ^***^*** | ***-5.9***  ***(-7.6, -4.1) ^***^*** |
| % sites CAL ≥ 4 mm | -30.9 (NE) | -21.6 (NE) | ***-10.1***  ***(-12.1, -8.0) ^***^*** | ***-8.2***  ***(-10.6, -5.7) ^***^*** | ***-6.2***  ***(-7.7, -4.7) ^***^*** | ***-6.3***  ***(-7.8, -4.8) ^***^*** | ***-3.3***  ***(-4.5, -2.2) ^***^*** | ***-3.1***  ***(-4.5, -1.7) ^***^*** |
| % sites CAL ≥ 5 mm | -21.5 (NE) | -19.3 (NE) | ***-5.9***  ***(-7.3, -4.6) ^***^*** | ***-4.7***  ***(-6.4, -3.1) ^***^*** | ***-3.8***  ***(-4.8, -2.8) ^***^*** | ***-3.9***  ***(-4.9, -2.9) ^***^*** | ***-1.7***  ***(-2.5, -0.9) ^***^*** | ***-1.6***  ***(-2.5, -0.6) ^**^*** |
| % sites CAL ≥ 6 mm | -16.9 (NE) | -11.1 (NE) | ***-3.3***  ***(-4.3, -2.4) ^***^*** | ***-2.5***  ***(-3.6, -1.4) ^***^*** | ***-2.3***  ***(-2.9, -1.6) ^***^*** | ***-2.2***  ***(-2.9, -1.5) ^***^*** | ***-1.1***  ***(-1.6, -0.6) ^***^*** | ***-0.9***  ***(-1.6, -0.4) ^**^*** |
| **UK Biobank** |  |  |  |  |  |  |  |  |
| **Bleeding gums (vs. healthy)** | ***0.8***  ***(0.7, 0.9) ^**^*** | ***0.8***  ***(0.7, 0.9) ^**^*** | ***0.3***  ***(0.08, 0.8) ^*^*** | ***0.2***  ***(0.06, 0.6) ^**^*** | ***0.8***  ***(0.7, 0.9) ^**^*** | ***0.7***  ***(0.6, 0.9) ^***^*** | 0.9  (0.8, 1.2) | 0.9  (0.8, 1.1) |
| **Painful gums (vs. healthy)** | ***0.7***  ***(0.5, 0.9) ^*^*** | 0.7  (0.5, 1.0) | 0.4  (0.1, 2.1) | 0.5  (0.1, 2.5) | ***0.7***  ***(0.5, 0.9) ^**^*** | ***0.5***  ***(0.4, 0.7) ^***^*** | 1.5  (0.9, 2.2) | 0.9  (0.6, 1.4) |
| **Loose teeth (vs. healthy)** | ***0.5***  ***(0.4, 0.7) ^***^*** | ***0.4***  ***(0.3, 0.6) ^***^*** | 0.2  (0.01, 1.8) | 0.2  (0.01, 2.8) | 0.8  (0.6, 1.0) | ***0.6***  ***(0.4, 0.7) ^***^*** | 1.1  (0.8, 1.5) | ***0.6***  ***(0.4, 0.9) ^*^*** |
| **Any periodontal disease (vs. healthy)** | ***0.6***  ***(0.3, 0.8) ^***^*** | ***0.3***  ***(0.3, 0.7) ^***^*** | 0.3  (0.01, 1.9) | 0.4  (0.2, 2.9) | 0.7  (0.6, 1.2) | ***0.7***  ***(0.4, 0.9)^**^*** | 1.2  (0.6, 1.6) | ***0.5***  ***(0.3, 0.8) ^*^*** |

Abbreviations. CAL, clinical attachment level; CI, confidence interval; MD, difference in means; mm, millimeter; NE, not estimable; OR, odds ratio; PPD, probing pocket depth; %, percentage.

^*^ p<0.05; ^**^ p<0.01; ^***^ p<0.001.

**Table S13**. Association between categories of healthy lifestyle score (2-3 vs. 0-1 healthy lifestyles) and periodontitis according to last dental visit and frequency of interproximal brushing (sensitivity analyses) in the NHANES cohort.

| **2-3 healthy lifestyles (vs. 0-1 healthy lifestyles) as exposure – OR/MD (95% CI)** | | | | | | | | | | | |
| --- | --- | --- | --- | --- | --- | --- | --- | --- | --- | --- | --- |
| **Outcomes** | **Last dental visit** | | | | **Frequency of interproximal brushing** | | | | | | |
|  | **6 months or less** | | **More than 6 months** | | **0 days/week** | | **1-6 days/week** | | **7 days/week** | | |
|  | ***Crude*** | ***Adjusted*** | ***Crude*** | ***Adjusted*** | ***Crude*** | ***Adjusted*** | ***Crude*** | ***Adjusted*** | ***Crude*** | ***Adjusted*** |  |
| **NHANES** |  |  |  |  |  |  |  |  |  |  |  |
| **Periodontitis** | ***0.4***  ***(0.3, 0.5) ^***^*** | ***0.4***  ***(0.3, 0.6) ^***^*** | ***0.5***  ***(0.4, 0.7) ^***^*** | ***0.4***  ***(0.3, 0.6) ^***^*** | ***0.5***  ***(0.4, 0.6) ^***^*** | ***0.4***  ***(0.3, 0.5) ^***^*** | ***0.6***  ***(0.5, 0.7) ^***^*** | ***0.6***  ***(0.4, 0.8) ^***^*** | ***0.5***  ***(0.4, 0.7) ^***^*** | ***0.5***  ***(0.3, 0.6) ^***^*** |  |
| **Periodontitis severity** |  |  |  |  |  |  |  |  |  |  |  |
| *Mild periodontitis* | 0.4  (0.09, 1.4) | 0.6  (0.2, 2.1) | 1.2  (0.4, 3.4) | 1.2  (0.4, 3.7) | ***0.3***  ***(0.2, 0.6) ^***^*** | ***0.2***  ***(0.1, 0.5) ^***^*** | 0.7  (0.4, 1.2) | 0.6  (0.3, 1.1) | 0.5  (0.2, 1.0) | 0.5  (0.2, 1.1) |  |
| *Moderate periodontitis* | ***0.4***  ***(0.3, 0.6) ^***^*** | ***0.3***  ***(0.2, 0.5) ^***^*** | ***0.3***  ***(0.2, 0.5) ^***^*** | ***0.2***  ***(0.1, 0.4) ^***^*** | ***0.3***  ***(0.2, 0.4) ^***^*** | ***0.1***  ***(0.01, 0.2) ^***^*** | ***0.4***  ***(0.3, 0.6) ^***^*** | ***03***  ***(0.2, 0.4) ^***^*** | ***0.5***  ***(0.4, 0.7) ^***^*** | ***0.3***  ***(0.2, 0.5) ^***^*** |  |
| *Severe periodontitis* | ***0.2***  ***(0.1, 0.3) ^***^*** | ***0.1***  ***(0.05, 0.2) ^***^*** | ***0.2***  ***(0.09, 0.3) ^***^*** | ***0.09***  ***(0.05, 0.2) ^***^*** | ***0.2***  ***(0.1, 0.3) ^***^*** | ***0.07***  ***(0.04, 0.1) ^***^*** | ***0.2***  ***(0.1, 0.3) ^***^*** | ***0.1***  ***(0.06, 0.2) ^***^*** | ***0.2***  ***(0.1, 0.3) ^***^*** | ***0.1***  ***(0.05, 0.2) ^***^*** |  |
| **PPD** |  |  |  |  |  |  |  |  |  |  |  |
| % sites PPD ≥ 4 mm | ***-1.3***  ***(-1.8, -0.8) ^***^*** | ***-0.9***  ***(-1.6, -0.4) ^**^*** | ***-1.3***  ***(-2.0, -0.6) ^**^*** | ***-1.3***  ***(-2.2, -0.5) ^***^*** | ***-2.2***  ***(-3.4, -1.1) ^***^*** | ***-1.9***  ***(-3.1, -0.7) ^**^*** | ***-1.3***  ***(-1.9, -0.6) ^***^*** | ***-0.9***  ***(-1.7, -0.2) ^*^*** | ***-1.9***  ***(-2.7, -1.1) ^***^*** | ***-1.6***  ***(-2.5, -0.8) ^***^*** |  |
| % sites PPD ≥ 5 mm | ***-0.3***  ***(-0.5, -0.08) ^**^*** | ***-0.2***  ***(-0.4, -0.009) ^*^*** | ***-0.4***  ***(-0.7, -0.06) ^*^*** | ***-0.4***  ***(-0.8, -0.02) ^***^*** | ***-0.8***  ***(-1.3, -0.2) ^*^*** | ***-0.6***  ***(-1.2, -0.04) ^*^*** | ***-0.4***  ***(-0.7, -0.09) ^*^*** | ***-0.3***  ***(-0.7, -0.003) ^*^*** | ***-0.6***  ***(-0.9, -0.2) ^**^*** | ***-0.5***  ***(-0.9, -0.09) ^*^*** |  |
| % sites PPD ≥ 6 mm | -0.05  (-0.2, 0.05) | -0.03  (-0.1, 0.09) | -0.04  (-0.2, 0.1) | -0.04  (-0.2, -0.1) | -0.3  (-0.6, 0.02) | -0.2  (-0.5, 0.09) | -0.06  (-0.2, 0.06) | -0.01  (-0.1, 0.1) | ***-0.2***  ***(-0.5, -0.001) ^*^*** | -0.2  (-0.5, 0.04) |  |
| **CAL** |  |  |  |  |  |  |  |  |  |  |  |
| % sites CAL ≥ 3 mm | ***-5.5***  ***(-7.5, -3.5) ^***^*** | ***-4.9***  ***(-7.2, -2.8) ^***^*** | ***-6.1***  ***(-8.0, -4.1) ^***^*** | ***-6.9***  ***(-9.1, -4.8) ^***^*** | ***-8.1***  ***(-10.3, -6.0) ^***^*** | ***-9.2***  ***(-11.5, -6.9) ^***^*** | ***-3.6***  ***(-5.3, -1.9) ^***^*** | ***-3.8***  ***(-5.8, -1.9) ^***^*** | ***-5.7***  ***(-8.1, -3.4) ^***^*** | ***-6.2***  ***(-8.4, .3.9) ^***^*** |  |
| % sites CAL ≥ 4 mm | ***-3.3***  ***(-4.5, -1.9) ^***^*** | ***-2.9***  ***(-4.3, -1.4) ^***^*** | ***-3.9***  ***(-5.4, -2.4) ^***^*** | ***-4.3***  ***(-6.0, -2.6) ^***^*** | ***-5.6***  ***(-7.4, -3.9) ^***^*** | ***-6.3***  ***(-8.3, -4.3) ^***^*** | ***-2.0***  ***(-3.2, -0.9) ^**^*** | ***-2.1***  ***(-3.3, -0.8) ^**^*** | ***-4.1***  ***(-5.9, -2.2) ^***^*** | ***-4.2***  ***(-6.2, -2.3) ^***^*** |  |
| % sites CAL ≥ 5 mm | ***-1.5***  ***(-2.2, -0.7) ^***^*** | ***-1.2***  ***(-2.0, -0.5) ^**^*** | ***-2.1***  ***(-3.2, -0.9) ^**^*** | ***-2.4***  ***(-3.7, -1.1) ^**^*** | ***-3.4***  ***(-4.7, -2.2) ^***^*** | ***-3.9***  ***(-5.4, -2.5) ^***^*** | ***-0.9***  ***(-1.5, -0.2) ^*^*** | ***-0.8***  ***(-1.6, -0.04) ^*^*** | ***-2.3***  ***(-3.6, -1.0) ^**^*** | ***-2.4***  ***(-3.8, -0.9) ^**^*** |  |
| % sites CAL ≥ 6 mm | ***-0.6***  ***(-0.9, -0.2) ^**^*** | ***-0.5***  ***(-0.8, -0.07) ^*^*** | ***-1.2***  ***(-1.9, -0.4) ^**^*** | ***-1.3***  ***(-2.2, -0.5) ^***^*** | ***-2.2***  ***(-3.0, -1.4) ^***^*** | ***-2.6***  ***(-3.5, -1.6) ^***^*** | -0.4  (-0.9, 0.05) | -0.3  (-0.8, 0.3) | ***-1.2***  ***(-2.0, -0.4) ^**^*** | ***-1.2***  ***(-2.1, -0.3) ^*^*** |  |

Abbreviations. CAL, clinical attachment level; CI, confidence interval; MD, difference in means; mm, millimeter; NE, not estimable; OR, odds ratio; PPD, probing pocket depth; %, percentage.

^*^ p<0.05; ^**^ p<0.01; ^***^ p<0.001.

**Table S14**. Association between categories of healthy lifestyle score (4-5 vs. 0-1 healthy lifestyles) and periodontitis according to last dental visit and frequency of interproximal brushing (sensitivity analyses) in the NHANES cohort.

| **4-5 healthy lifestyles (vs. 0-1 healthy lifestyles) as exposure – OR/MD (95% CI)** | | | | | | | | | | |
| --- | --- | --- | --- | --- | --- | --- | --- | --- | --- | --- |
| **Outcomes** | **Last dental visit** | | | | **Frequency of interproximal brushing** | | | | | |
|  | **6 months or less** | | **More than 6 months** | | **0 days/week** | | **1-6 days/week** | | **7 days/week** | |
|  | ***Crude*** | ***Adjusted*** | ***Crude*** | ***Adjusted*** | ***Crude*** | ***Adjusted*** | ***Crude*** | ***Adjusted*** | ***Crude*** | ***Adjusted*** |
| **NHANES** |  |  |  |  |  |  |  |  |  |  |
| **Periodontitis** | ***0.4***  ***(0.3, 0.5) ^***^*** | ***0.4***  ***(0.2, 0.6) ^***^*** | ***0.3***  ***(0.2, 0.5) ^***^*** | ***0.2***  ***(0.2, 0.4) ^***^*** | ***0.3***  ***(0.2, 0.3) ^***^*** | ***0.2***  ***(0.1, 0.2) ^***^*** | ***0.4***  ***(0.3, 0.5) ^***^*** | ***0.4***  ***(0.3, 0.6) ^***^*** | ***0.4***  ***(0.3, 0.6) ^***^*** | ***0.4***  ***(0.3, 0.5) ^***^*** |
| **Periodontitis severity** |  |  |  |  |  |  |  |  |  |  |
| *Mild periodontitis* | 0.3  (0.09, 1.2) | 0.5  (0.2, 2.1) | 1.0  (0.5, 2.9) | 1.2  (0.4, 3.9) | ***0.2***  ***(0.1, 0.4) ^***^*** | ***0.2***  ***(0.1, 0.5) ^***^*** | 0.5  (0.3, 1.1) | 0.5  (0.2, 1.0) | ***0.4***  ***(0.2, 0.9) ^*^*** | 0.4  (0.1, 1.0) |
| *Moderate periodontitis* | ***0.3***  ***(0.2, 0.6) ^***^*** | ***0.2***  ***(0.1, 0.4) ^***^*** | ***0.3***  ***(0.2, 0.5) ^***^*** | ***0.2***  ***(0.1, 0.5) ^***^*** | ***0.2***  ***(0.1, 0.3) ^***^*** | ***0.1***  ***(0.09, 0.2) ^***^*** | ***0.4***  ***(0.2, 0.6) ^***^*** | ***0.2***  ***(0.1, 0.5) ^***^*** | ***0.4***  ***(0.2, 0.7) ^**^*** | ***0.2***  ***(0.1, 0.5) ^***^*** |
| *Severe periodontitis* | ***0.1***  ***(0.03, 0.2) ^***^*** | ***0.09***  ***(0.02, 0.2) ^***^*** | ***0.1***  ***(0.04, 0.3) ^***^*** | ***0.09***  ***(0.04, 0.2) ^***^*** | ***0.1***  ***(0.03, 0.2) ^***^*** | ***0.07***  ***(0.04, 0.1) ^***^*** | ***0.1***  ***(0.04, 0.2) ^***^*** | ***0.1***  ***(0.02, 0.2) ^***^*** | ***0.1***  ***(0.06, 0.2) ^***^*** | ***0.1***  ***(0.02, 0.3) ^***^*** |
| **PPD** |  |  |  |  |  |  |  |  |  |  |
| % sites PPD ≥ 4 mm | ***-1.7***  ***(-2.2, -1.2) ^***^*** | ***-1.3***  ***(-1.9, -0.8) ^***^*** | ***-2.7***  ***(-3.5, -1.8) ^***^*** | ***-2.5***  ***(-3.4, -1.6) ^***^*** | ***-3.2***  ***(-4.3, -2.0) ^***^*** | ***-2.4***  ***(-3.6, -1.2) ^**^*** | ***-2.3***  ***(-2.9, -1.6) ^***^*** | ***-1.7***  ***(-2.4, -0.9) ^***^*** | ***-2.7***  ***(-3.5, -1.9) ^***^*** | ***-2.2***  ***(-3.1, -1.3) ^***^*** |
| % sites PPD ≥ 5 mm | ***-0.4***  ***(-0.6, -0.2) ^***^*** | ***-0.3***  ***(-0.5, -0.1) ^**^*** | ***-0.9***  ***(-1.2, -0.6) ^***^*** | ***-0.8***  ***(-1.2, -0.4) ^***^*** | ***-0.9***  ***(-1.5, -0.4) ^**^*** | -0.5  (-1.1, 0.05) | ***-0.7***  ***(-1.1, -0.4) ^***^*** | ***-0.6***  ***(-0.9, -0.2) ^**^*** | ***-0.9***  ***(-1.3, -0.5) ^***^*** | ***-0.7***  ***(-1.1, -0.3) ^**^*** |
| % sites PPD ≥ 6 mm | -0.07  (-0.2, 0.02) | -0.04  (-0.2, 0.06) | ***-0.2***  ***(-0.4, -0.05) ^*^*** | ***-0.2***  ***(-0.4, -0.02) ^*^*** | ***-0.3***  ***(-0.6, -0.02) ^*^*** | -0.1  (-0.4, 0.2) | ***-0.2***  ***(-0.3, -0.06) ^**^*** | -0.09  (-0.2, 0.01) | ***-0.3***  ***(-0.6, -0.09) ^**^*** | ***-0.3***  ***(-0.6, -0.03) ^*^*** |
| **CAL** |  |  |  |  |  |  |  |  |  |  |
| % sites CAL ≥ 3 mm | ***-6.5***  ***(-8.7, -4.3) ^***^*** | ***-6.0***  ***(-8.6, -3.5) ^***^*** | ***-10.6***  ***(-12.9, -8.2) ^***^*** | ***-10.6***  ***(-13.4, -7.8) ^***^*** | ***-11.1***  ***(-13.5, -8.7) ^***^*** | ***-11.9***  ***(-14.9, -8.9) ^***^*** | ***-6.7***  ***(-8.5, -4.9) ^***^*** | ***-6.1***  ***(-8.3, -3.9) ^***^*** | ***-7.5***  ***(-9.8, -5.3) ^***^*** | ***-7.8***  ***(-10.1, -5.4) ^***^*** |
| % sites CAL ≥ 4 mm | ***-4.0***  ***(-5.3, -2.7) ^***^*** | ***-3.5***  ***(-5.1, -1.9) ^***^*** | ***-6.7***  ***(-8.4, -4.9) ^***^*** | ***-6.4***  ***(-8.5, -4.2) ^***^*** | ***-7.5***  ***(-9.4, -5.5) ^***^*** | ***-7.7***  ***(-10.2, -5.2) ^***^*** | ***-3.7***  ***(-4.8, -2.6) ^***^*** | ***-3.3***  ***(-4.5, -2.0) ^***^*** | ***-5.5***  ***(-7.4, -3.5) ^***^*** | ***-5.5***  ***(-7.6, -3.4) ^***^*** |
| % sites CAL ≥ 5 mm | ***-1.9***  ***(-2.6, -1.3) ^***^*** | ***-1.7***  ***(-2.4, -0.9) ^***^*** | ***-3.8***  ***(-4.9, -2.6) ^***^*** | ***-3.6***  ***(-5.2, -2.1) ^***^*** | ***-4.5***  ***(-6.0, -3.1) ^***^*** | ***-4.8***  ***(-6.6, -2.9) ^***^*** | ***-1.9***  ***(-2.5, -1.2) ^***^*** | ***-1.6***  ***(-2.3, -0.9) ^***^*** | ***-3.2***  ***(-4.6, -1.8) ^***^*** | ***-3.2***  ***(-4.8, -1.7) ^***^*** |
| % sites CAL ≥ 6 mm | ***-0.9***  ***(-1.3, -0.5) ^***^*** | ***-0.7***  ***(-1.1, -0.3) ^**^*** | ***-2.2***  ***(-2.9, -1.5) ^***^*** | ***-2.1***  ***(-3.0, -1.2) ^***^*** | ***-2.9***  ***(-3.9, -2.1) ^***^*** | ***-3.2***  ***(-4.4, -1.9) ^***^*** | ***-0.9***  ***(-1.4, -0.6) ^***^*** | ***-0.7***  ***(-1.2, -0.3) ^**^*** | ***-1.7***  ***(-2.6, -0.8) ^***^*** | ***-1.7***  ***(-2.7, -0.7) ^**^*** |

Abbreviations. CAL, clinical attachment level; CI, confidence interval; MD, difference in means; mm, millimeter; NE, not estimable; OR, odds ratio; PPD, probing pocket depth; %, percentage.

^*^ p<0.05; ^**^ p<0.01; ^***^ p<0.001.

**Table S15**. Correlation between markers of systemic inflammation and periodontitis in both cohorts.

|  | **WBC** | **CRP** | **Neutrophils count** |
| --- | --- | --- | --- |
| **NHANES** |  |  |  |
| Periodontitis |  |  |  |
| *R/F value* | 5.01 | 50.01 | 188.85 |
| *p-value* | ***0.025^*^*** | ***<0.001^***^*** | ***<0.001^***^*** |
| % sites PPD ≥ 4 mm |  |  |  |
| *R/F value* | 0.068 | 0.097 | 0.082 |
| *p-value* | ***<0.001^***^*** | ***<0.001^***^*** | ***<0.001^***^*** |
| % sites PPD ≥ 5 mm |  |  |  |
| *R/F value* | 0.035 | 0.078 | 0.047 |
| *p-value* | ***<0.001^***^*** | ***<0.001^***^*** | ***<0.001^***^*** |
| % sites PPD ≥ 6 mm |  |  |  |
| *R/F value* | 0.020 | 0.077 | 0.028 |
| *p-value* | ***0.005^*^*** | ***<0.001^***^*** | ***<0.001^***^*** |
| % sites CAL ≥ 3 mm |  |  |  |
| *R/F value* | 0.062 | 0.111 | 0.099 |
| *p-value* | ***<0.001^***^*** | ***<0.001^***^*** | ***<0.001^***^*** |
| % sites CAL ≥ 4 mm |  |  |  |
| *R/F value* | 0.059 | 0.094 | 0.086 |
| *p-value* | ***<0.001^***^*** | ***<0.001^***^*** | ***<0.001^***^*** |
| % sites CAL ≥ 5 mm |  |  |  |
| *R/F value* | 0.049 | 0.087 | 0.069 |
| *p-value* | ***<0.001^***^*** | ***<0.001^***^*** | ***<0.001^***^*** |
| % sites CAL ≥ 6 mm |  |  |  |
| *R/F value* | 0.036 | 0.085 | 0.051 |
| *p-value* | ***<0.001^***^*** | ***<0.001^***^*** | ***<0.001^***^*** |
| **UK Biobank** |  |  |  |
| **Bleeding gums** |  |  |  |
| *F value* | 87.85 | 47.68 | 71.77 |
| *p-value* | ***<0.001^***^*** | ***<0.001^***^*** | ***<0.001^***^*** |
| **Painful gums** |  |  |  |
| *F value* | 114.79 | 63.96 | 54.51 |
| *p-value* | ***<0.001^***^*** | ***<0.001^***^*** | ***<0.001^***^*** |
| **Loose teeth** |  |  |  |
| *F value* | 214.39 | 72.27 | 148.77 |
| *p-value* | ***<0.001^***^*** | ***<0.001^***^*** | ***<0.001^***^*** |

**References**

Abdullah Said, M., Verweij, N., & Van Der Harst, P. (2018). Associations of combined genetic and lifestyle risks with incident cardiovascular disease and diabetes in the UK biobank study. *JAMA Cardiology*, *3*(8), 693–702. https://doi.org/10.1001/jamacardio.2018.1717

Hirshkowitz, M., Whiton, K., Albert, S. M., Alessi, C., Bruni, O., DonCarlos, L., Hazen, N., Herman, J., Katz, E. S., Kheirandish-Gozal, L., Neubauer, D. N., O’Donnell, A. E., Ohayon, M., Peever, J., Rawding, R., Sachdeva, R. C., Setters, B., Vitiello, M. V., Ware, J. C., & Adams Hillard, P. J. (2015). National sleep foundation’s sleep time duration recommendations: Methodology and results summary. *Sleep Health*, *1*(1), 40–43. https://doi.org/10.1016/j.sleh.2014.12.010

Janssen, I., Carson, V., Lee, I. M., Katzmarzyk, P. T., & Blair, S. N. (2013). Years of life gained due to leisure-time physical activity in the U.S. *American Journal of Preventive Medicine*, *44*(1), 23–29. https://doi.org/10.1016/j.amepre.2012.09.056

Manea, L., Gilbody, S., & McMillan, D. (2012). Optimal cut-off score for diagnosing depression with the Patient Health Questionnaire (PHQ-9): A meta-analysis. *CMAJ. Canadian Medical Association Journal*, *184*(3). https://doi.org/10.1503/cmaj.110829

Ricci, C., Schutte, A. E., Schutte, R., Smuts, C. M., & Pieters, M. (2020). Trends in alcohol consumption in relation to cause-specific and all-cause mortality in the United States: A report from the NHANES linked to the US mortality registry. *American Journal of Clinical Nutrition*, *111*(3), 580–589. https://doi.org/10.1093/ajcn/nqaa008

Zhang, Y. B. o., Chen, C., Pan, X. F., Guo, J., Li, Y., Franco, O. H., Liu, G., & Pan, A. (2021). Associations of healthy lifestyle and socioeconomic status with mortality and incident cardiovascular disease: Two prospective cohort studies. *The BMJ*, *373*. https://doi.org/10.1136/bmj.n604

Zhang, Y. B., Pan, X. F., Chen, J., Cao, A., Xia, L., Zhang, Y., Wang, J., Li, H., Liu, G., & Pan, A. (2021). Combined lifestyle factors, all-cause mortality and cardiovascular disease: A systematic review and meta-analysis of prospective cohort studies. In *Journal of Epidemiology and Community Health* (Vol. 75, Issue 1, pp. 92–99). BMJ Publishing Group. https://doi.org/10.1136/jech-2020-214050

Zhang, Y., Pan, X. F., Chen, J., Xia, L., Cao, A., Zhang, Y., Wang, J., Li, H., Yang, K., Guo, K., He, M., & Pan, A. (2020). Combined lifestyle factors and risk of incident type 2 diabetes and prognosis among individuals with type 2 diabetes: a systematic review and meta-analysis of prospective cohort studies. In *Diabetologia* (Vol. 63, Issue 1, pp. 21–33). Springer. https://doi.org/10.1007/s00125-019-04985-9
